# Surface slicks are pelagic nurseries for diverse ocean fauna

**DOI:** 10.1038/s41598-021-81407-0

**Published:** 2021-02-04

**Authors:** Jonathan L. Whitney, Jamison M. Gove, Margaret A. McManus, Katharine A. Smith, Joey Lecky, Philipp Neubauer, Jana E. Phipps, Emily A. Contreras, Donald R. Kobayashi, Gregory P. Asner

**Affiliations:** 1 Joint Institute for Marine and Atmospheric Research, University of Hawai‘i at Mānoa, Honolulu, HI 96822 USA; 2 Pacific Islands Fisheries Science Center, National Oceanic and Atmospheric Administration, Honolulu, HI 96818 USA; 3 Department of Oceanography, University of Hawai‘i at Mānoa, Honolulu, HI 96822 USA; 4 Lynker Technologies LLC, Marine, Ocean, and Coastal Science and Information Group, Leesburg, VA 20175 USA; 5 Dragonfly Data Science, 158 Victoria St, Level 4, Te Aro, Wellington, 6011 New Zealand; 6 Center for Global Discovery and Conservation Science, Arizona State University, Tempe, AZ 85281 USA

**Keywords:** Biooceanography, Coral reefs, Fisheries, Biodiversity, Ecosystem ecology, Ecology, Community ecology, Food webs, Ocean sciences, Marine biology, Zoology, Ichthyology

## Abstract

Most marine animals have a pelagic larval phase that develops in the coastal or open ocean. The fate of larvae has profound effects on replenishment of marine populations that are critical for human and ecosystem health. Larval ecology is expected to be tightly coupled to oceanic features, but for most taxa we know little about the interactions between larvae and the pelagic environment. Here, we provide evidence that surface slicks, a common coastal convergence feature, provide nursery habitat for diverse marine larvae, including > 100 species of commercially and ecologically important fishes. The vast majority of invertebrate and larval fish taxa sampled had mean densities 2–110 times higher in slicks than in ambient water. Combining in-situ surveys with remote sensing, we estimate that slicks contain 39% of neustonic larval fishes, 26% of surface-dwelling zooplankton (prey), and 75% of floating organic debris (shelter) in our 1000 km^2^ study area in Hawai‘i. Results indicate late-larval fishes actively select slick habitats to capitalize on concentrations of diverse prey and shelter. By providing these survival advantages, surface slicks enhance larval supply and replenishment of adult populations from coral reef, epipelagic, and deep-water ecosystems. Our findings suggest that slicks play a critically important role in enhancing productivity in tropical marine ecosystems.

Life for many marine organisms begins near the ocean surface. Buoyant eggs hatch into planktonic larvae that develop and disperse in the ocean for weeks to months before transitioning into juveniles and eventually finding suitable adult habitat^1^. The pelagic larval stage connects populations and serves as a source of new adults. Oceanic processes affecting the fate of larvae have profound impacts on population replenishment, connectivity, and ecosystem structure^2^. Despite the importance of this life stage, we have limited knowledge of the ecology and behaviour of larvae. Understanding the biophysical interactions that govern larval fish survival and transport is essential for predicting and managing marine ecosystems, as well as the fisheries they support^3,4^.

The distribution of prey and predators in the ocean is considerably patchy^5,6^. Larval survival depends strongly on prey availability, predation, and transport to suitable habitat, all of which are influenced by ocean conditions^7^. Ocean processes that drive convergent flow, such as fronts, internal waves, and eddies, can structure plankton, enhance overlap of predators and prey, and influence larval dispersal^8–15^. Convergent features can also lead to a cascade of effects that ultimately drive food web structure and increase ecosystem productivity^16^.

Slicks are meandering lines of smooth water on the ocean surface that are ubiquitous coastal features around the world^17^. While a variety of mechanisms can cause slick formation, including tidal and headland fronts, in Hawaii’s coastal waters slicks form predominantly as a consequence of subsurface waves, called internal waves^18^. Internal wave slicks are generated when internal waves interact with steep seafloor topography and drive areas of convergence and divergence at the ocean surface^19^ (see Supplementary Fig. S1). The build-up of organic material (surfactants) at the surface modifies surface tension causing a smooth, oil slick-like appearance^20^ (Fig. 1d). The convergent flow can accumulate dense aggregations of plankton including larval fish and invertebrates at or below the ocean surface^8,21–26^. Historical research on biological accumulation in surface slicks has largely been restricted to temperate systems (but see Kingsford et al.^25^). Before this contribution, the ecological importance of surface slicks to developing larvae in tropical and subtropical marine ecosystems was largely unknown.

**Figure 1:**
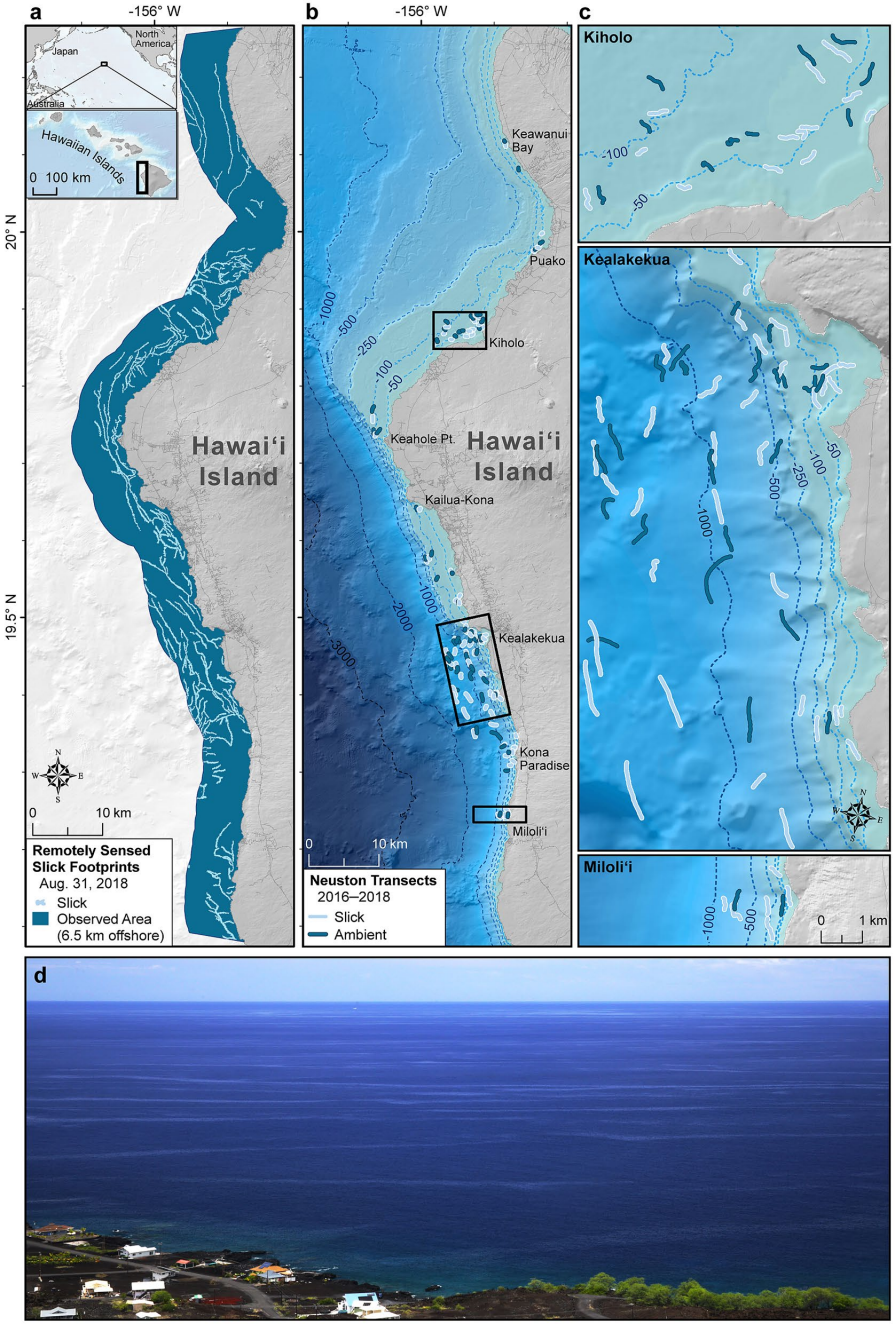
Images of surface slicks and study site along the west coast of Hawai‘i Island. (**a**) Remotely sensed observations of surface slicks for 31 August 2018, utilizing Planet Dove satellite images and a mapping approach that classifies slicks using the contrast between surface texture of slicks and regular ambient seawater. The spatial extent of remote sensing detection of slicks is shown as blue shaded regions and constrained to the spatial extent of our neuston tows (≤ 6.5 km). (**b**) Location of neuston tows (*n* = 134) in both slicks (light blue) and ambient water (dark blue), (**c**), highlighting site level detail. (**d**) Photograph of surface slicks along the coast of West Hawai**‘**i. Slicks are formed by a variety of mechanisms, but primarily when internal waves interact with steep seafloor topography and create convergent currents at the surface that can appear as areas of smooth water. In convergent flows (see Supplementary Fig. S1), the build-up of insoluble organic matter at the surface (primarily from plankton) creates a biogenic marine film that dampens small-scale waves, thereby causing a significant reduction of the glittering effect of sun beams^20^. The resulting surface manifestation appears as a smooth, oily slick and can be visible even from satellite images (**a**). Maps were produced with ArcGIS Desktop 10.6 software (https://desktop.arcgis.com/).

Nursery habitats contribute disproportionately to the replenishment of adult populations by supporting increased juvenile densities, faster growth, higher survival, or the successful movement of larvae and juveniles to adult habitats, when compared to ambient ocean habitats^27^. Most examples of marine nurseries are shallow-water, coastal estuaries (e.g., mangroves and seagrass meadows) where juveniles grow to subadults then move to adult habitat offshore or to nearby reefs^28,29^. Few studies have evaluated how ocean features may serve as nurseries for pelagic larval stages of fishes and invertebrates (e.g., mesoscale eddies^14,30–32^), and to our knowledge, none have assessed the nursery role of surface slicks.

We surveyed neustonic zooplankton and ichthyoplankton communities inside and outside of surface slicks in the coastal waters along the west coast of the Island of Hawai‘i (hereafter “West Hawai‘i”)(see “Methods”—“Study area” for detailed description). Internal wave generation is common in Hawai‘i^33^, and surface slicks are prevalent features across the West Hawai‘i coastline (Fig. 1a)^34^. In addition, due to the region’s complex bathymetry, multiple marine habitats are compressed towards shore and early life stages from diverse communities of coral reef, epipelagic, and deep-water fishes overlap in close proximity to the islands^35,36^. This combination of high biodiversity and prominence of surface slicks in the region^34,37^ makes it an ideal model system in which to evaluate ecological interactions.

## Results

**Figure 2:**
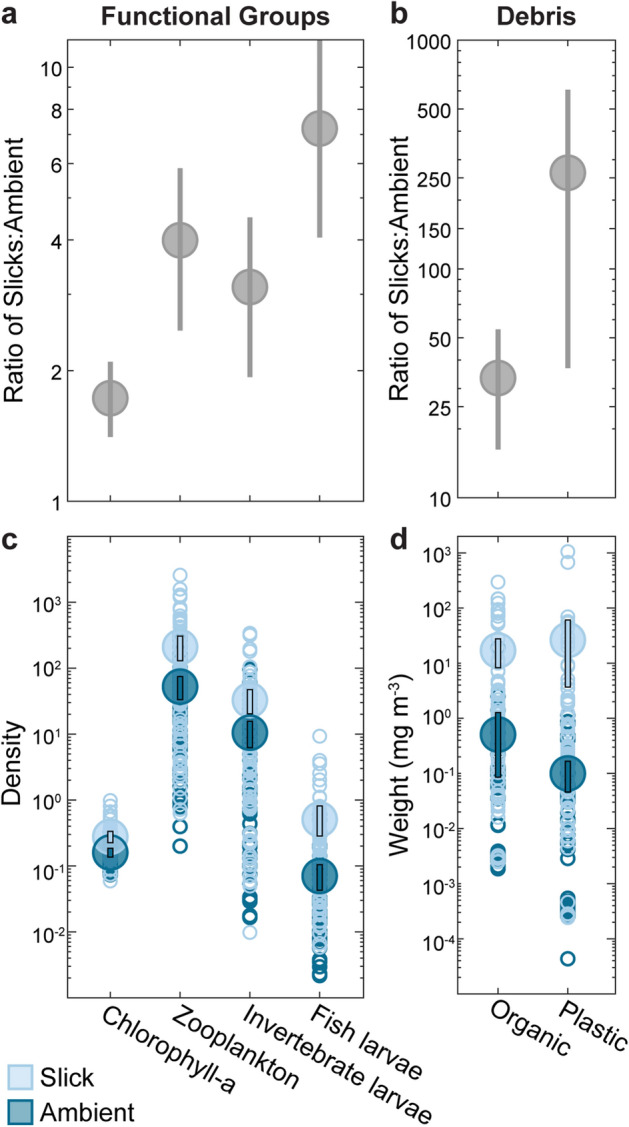
Densities and response ratios of major planktonic functional groups and debris in surface slicks compared to ambient waters. (**a**) Ratio of mean density in slicks to mean density in ambient water ± 95% bootstrap intervals (BI) for major planktonic functional groups (Supplementary Table S1). (**b**) Ratio of mean dry weight in slicks to mean dry weight in ambient water ± 95% BI for organic and plastic debris. (**c**) Mean (± 95% BI) densities of total zooplankton (i.e., including larvae and holozooplankton; individuals m^−3^), invertebrate larvae (individuals m^−3^), fish larvae (individuals  m^−3^) and concentration of chlorophyll-*a*, (mg m^−3^, a proxy for phytoplankton) (Supplementary Table S1). (**d**) Mean (± 95% BI) dry weight (mg m^−3^) of floating organic and plastic debris. Open circles indicate individual neuston tow samples in slicks (light blue) and ambient waters (dark blue) as follows: chlorophyll-*a* (*n* = 44 slick, *n* = 27 ambient); zooplankton, invertebrate larvae, fish larvae, organic debris, plastic debris (*n* = 80 slick, *n* = 54 ambient).

We conducted 134 surface-neuston tows, including 80 inside surface slicks and 54 in areas outside of slicks, hereafter referred to as “ambient waters”, in the coastal waters of West Hawai‘i (Fig. 1). Mean densities of chlorophyll-*a* (proxy for phytoplankton), zooplankton, larval invertebrates, and larval fishes were 1.7, 4.0, 3.1, and 7.2 times higher, respectively, in surface slicks compared to ambient waters (P($\bar{d}$_*slick*_>$\bar{d}$_*ambient*_) > 0.98; Fig. 2; Supplementary Table S1; see also Ref.^34^). P($\bar{d}$_*slick*_>$\bar{d}$_*ambient*_) represents the empirical probability that the mean density ($\bar{d}$ inside slicks is greater than in ambient water (see “Methods—Statistical analyses”). The slick convergence zones create highly heterogeneous habitat that structures the neustonic community into dense bands.

We found that the mass of all floating debris was 71.0 times higher in slicks compared to ambient waters (mean dry weight: 43.31 mg m^−3^ slicks; 0.61 mg m^−3^ ambient; P($\bar{d}$_*slick*_>$\bar{d}$_*ambient*_) ≥ 0.9999; Fig. 2; Supplementary Table S1). By dry weight, the majority of floating debris was plastic (61.1%), while the remaining 38.9% was naturally occurring organic debris consisting of macroalgae, plant matter (e.g., leaves, twigs, seeds), and animal matter (e.g., feathers, arthropod molts). The mass of organic floating debris was 34.3 times greater in slicks compared to ambient waters (mean dry weight: 16.96 mg m^−3^ slicks; 0.51 mg m^−3^ ambient; P($\bar{d}$_*slick*_>$\bar{d}$_*ambient*_) ≥ 0.9999). The dry mass of plastic debris, which consisted of primarily polyethylene and polypropylene fragments and microfibers^34^, was 263.3 times higher in slicks than in ambient water (mean: 26.33 mg m^−3^ slicks; 0.10 mg m^−3^ ambient; P($\bar{d}$_*slick*_>$\bar{d}$_*ambient*_) ≥ 0.9999).

**Figure 3:**
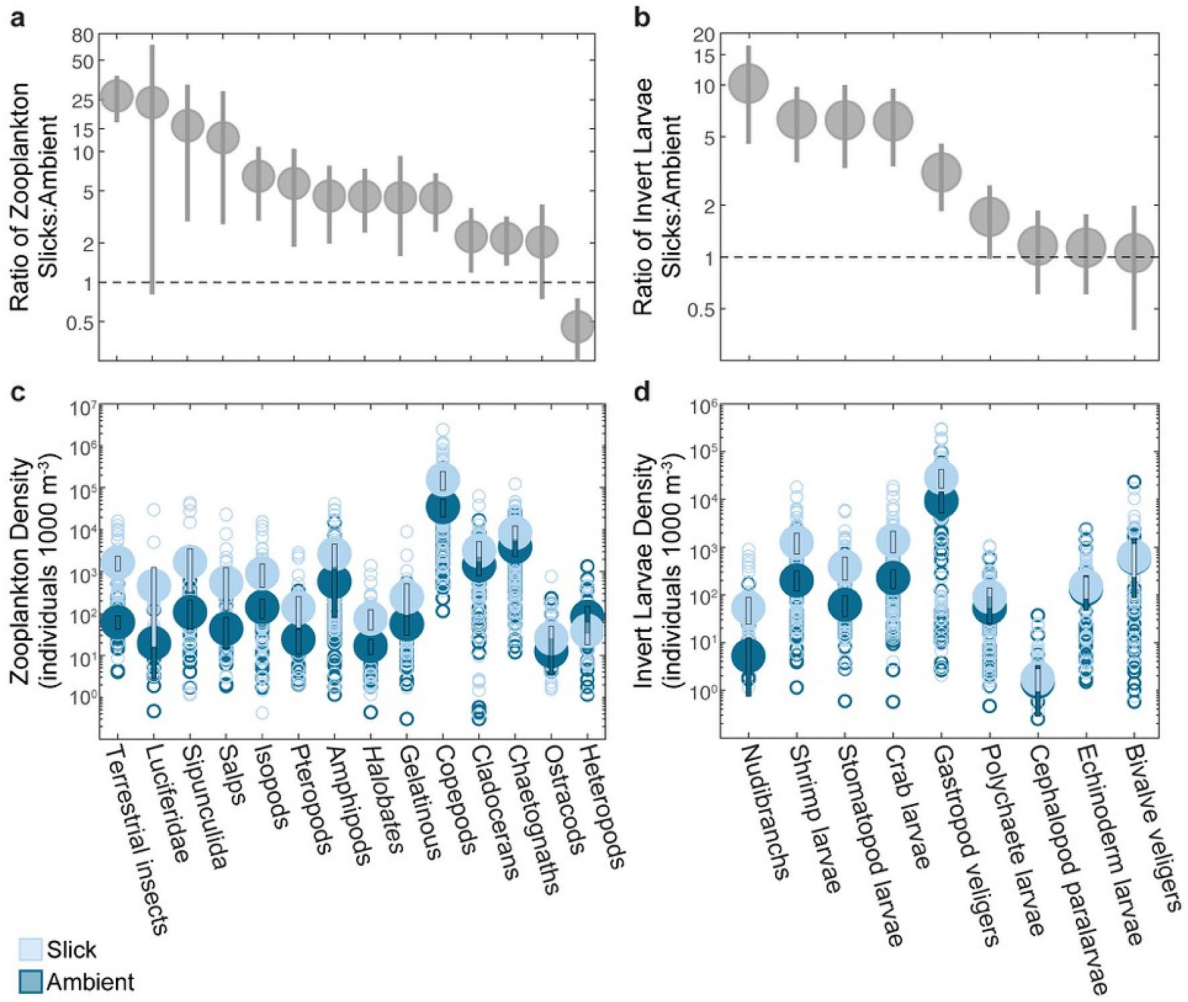
Densities and response ratios of zooplankton groups in surface slicks compared to ambient waters. (**a**,**b**) Ratio of mean density in slicks to mean density in ambient water ± 95% bootstrap intervals for (**a**) zooplankton (i.e., holoplankton) and (**b**) invertebrate larvae (i.e., meroplankton). The dashed line at Y = 1 indicates equal densities in both habitats. (**c**,**d**) Mean (± 95% bootstrap intervals) densities of (**c**) zooplankton and (**d**) invertebrate larvae (individuals 1000 m^−3^). Open circles indicate individual neuston tow samples in slicks (light blue) and ambient waters (dark blue) as follows: zooplankton, invertebrate larvae (*n* = 80 slick, *n* = 54 ambient).

The invertebrate zooplankton community in waters off West Hawai‘i was highly diverse. We recorded and identified 23 distinct zooplankton taxa in our study, 78% (18/23) of which had mean densities that were between 2.0 and 26.6 times higher in slicks than in ambient water (Fig. 3, Supplementary Table S2). The majority (56%) of larval invertebrate taxa had at least three times greater density in surface slicks than in ambient water (P($\bar{d}$_*slick*_>$\bar{d}$_*ambient*_) > 0.99). This includes larval nudibranchs (10.1 times higher in slicks), shrimps (6.3 times), stomatopods (6.2 times), crabs (6.1 times), and gastropods (3.1 times) (Fig. 3). Multiple larval stages of shrimp (zoea and decapodite) and crab larvae (zoea and megalops) were also highly concentrated in slicks, with mean densities between 5.8 and 7.6 times those in ambient waters (Supplementary Table S2).

We provide the first evidence that marine water striders (*Halobates* spp.) are strongly associated with surface slicks. These pleustonic insects (*Halobates hawaiiensis* and *H. sericeus*) were 6.6 times more abundant in slicks (Fig. 3; Supplementary Table S2). *Halobates* spp. subsist principally on terrestrial insects (e.g., bees, beetles, moths) blown out to sea^38^, which were 26.6 times more concentrated in slicks than in ambient waters. *Halobates* are themselves prey to a number of larval and juvenile fishes^39^ found in slicks (Supplementary Note 1).

Comparing correlations in abundance, we found that slick habitats exhibit enhanced overlap between multiple prey groups and larval fish predators (Supplementary Fig. S2). In ambient waters, larval fish abundance was significantly correlated with just two prey taxa (*r* = 0.29–0.44; *P* < 0.05). However, in slicks, larval fish abundance was significantly correlated with seven prey taxa (*r* = 0.25–0.62; *P* < 0.05). Correlation coefficients in slicks were higher in 11/12 larval fish pairwise comparisons and were on average 3 times higher (mean *r* = 0.30 slicks, *r* = 0.10 ambient).

**Figure 4:**
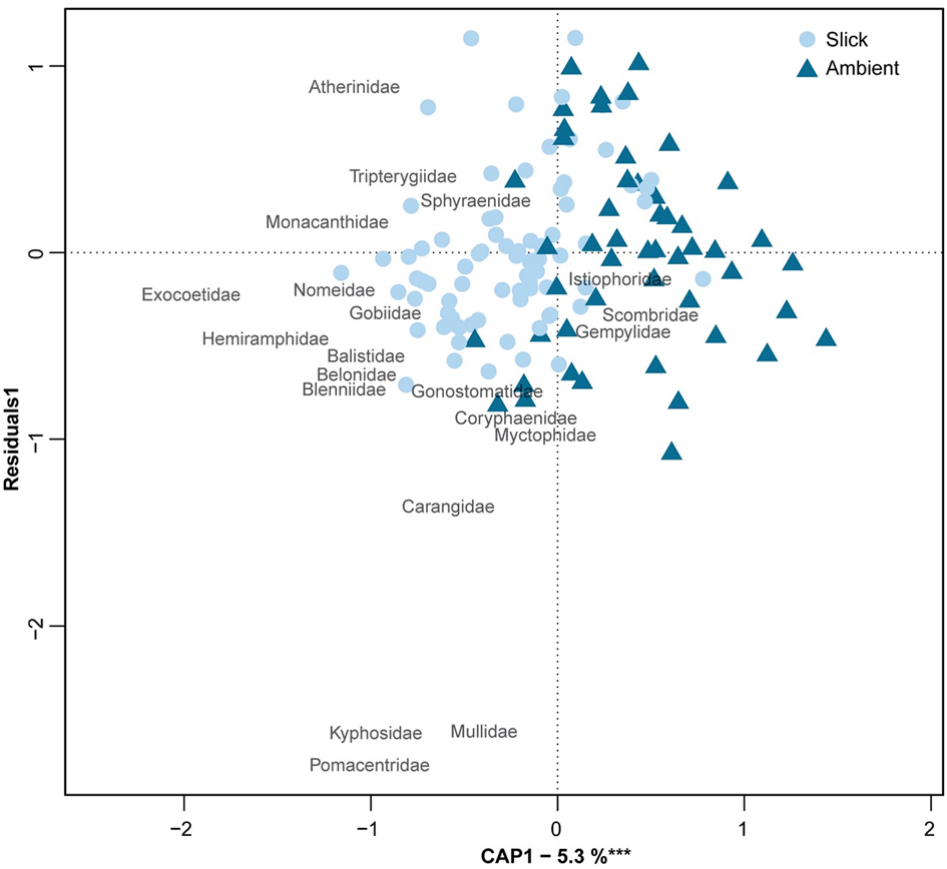
Redundancy analysis ordination plot showing the influence of surface slicks on neustonic ichthyoplankton community using Bray–Curtis distance. Transects representing neuston tows in surface slicks (light blue circles, *n* = 80) and in ambient waters (dark blue triangles, *n* = 54) are plotted onto the first constrained axis (CAP1) and first residual axis (Residuals1). The ‘species’ scores (represented by family names) are scaled to site scores and the distances among them approximate Euclidean distance. *** indicates CAP1 significance level (*P* < 0.001).

We identified and measured 13,217 fish specimens representing more than 118 species from 54 families (Supplementary Tables S3, S4). Ninety-five percent of individuals were identified to at least family, 77% to genus, and 51% to species. We found that surface slicks contained 41% higher mean fish diversity (Shannon’s: H′ = 1.58 slick; H′ = 1.12 ambient) and 2.2 times the taxonomic richness (S = 11.53 vs. S = 5.13) than ambient waters (P($\bar{d}$_*slick*_>$\bar{d}$_*ambient*_) ≥ 0.9999). In slicks, we recorded a total of 112 unique larval fish taxa representing 54 families. By comparison, ambient water contained 50% fewer unique taxa (57) and 39% fewer families (33) (Supplementary Tables S3, S4). Using a distance-based redundancy analysis, we found that the larval fish community structure was significantly different in slicks compared to ambient waters (*F*_1,122_ = 4.53, *P* < 0.001; Fig. 4). For example, in ambient water, goatfishes (Mullidae) were the dominant family (28.7%), whereas in slicks, the flyingfishes (Exocoetidae) were dominant, representing nearly half (43%) of all individuals. Therefore, the observed differences in fish diversity are driven by a combination of shifts in relative abundance, numerical dominance, and taxonomic richness.

**Figure 5:**
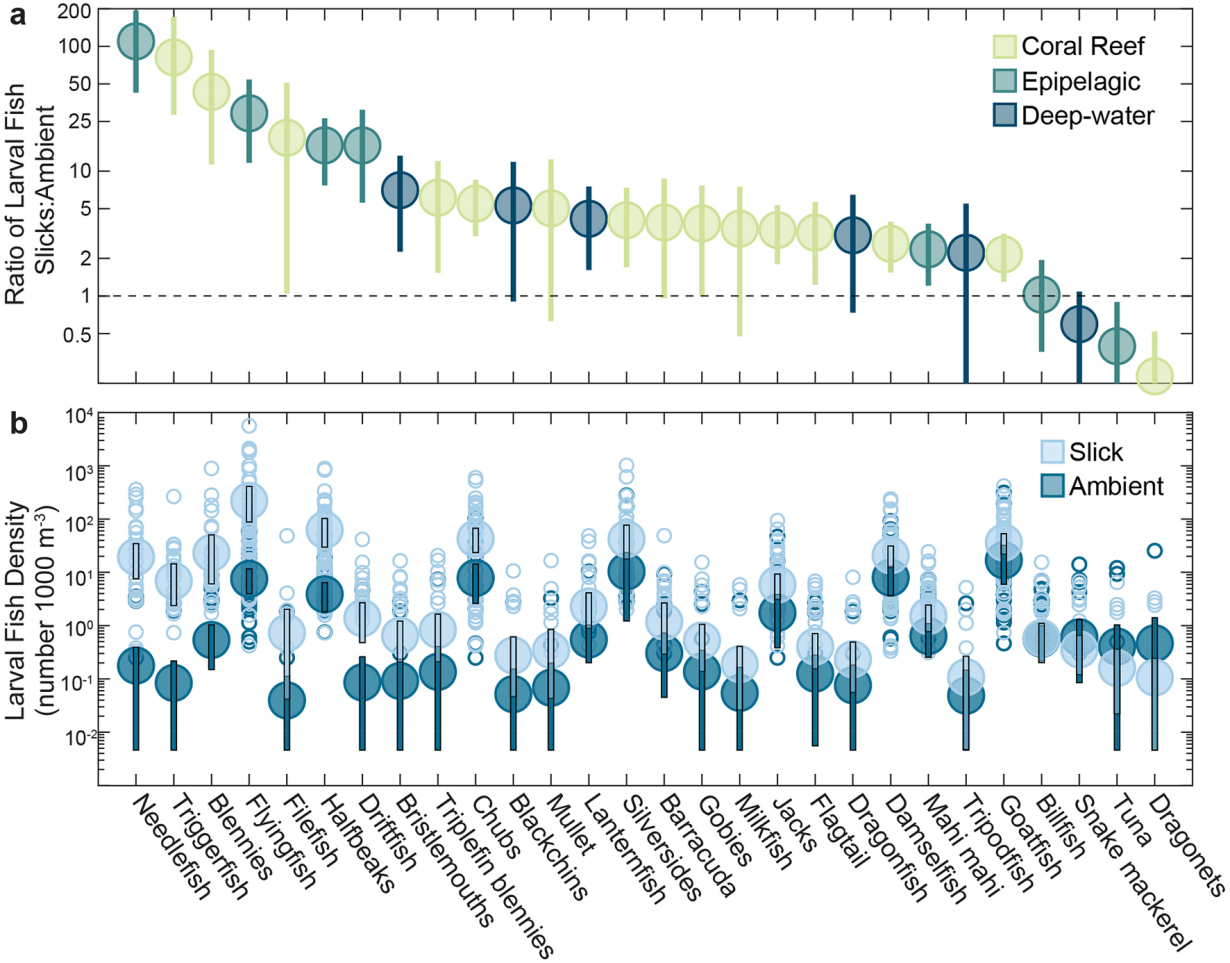
Accumulation ratios and densities of larval fish families in surface slicks compared to ambient waters. (**a**) Ratio of mean density in slicks to mean density in ambient water ± 95% bootstrap intervals for larval fish families from three natal habitats (Coral Reefs, Epipelagic and Deep-water). The dashed line at Y = 1 indicates equal densities in both habitats. Five additional reef fish families: pufferfishes (Tetraodontidae), cardinalfishes (Apogonidae), frogfishes (Antennariidae), squirrelfishes (Holocentridae), and surf sardines (Isonidae) that were found exclusively in slicks were excluded from the plot due to undefined ratios. (**b**) Mean (± 95% bootstrap intervals) densities of larval fish families from three natal habitats (Coral Reef, Epipelagic and Deep-water) in slicks (light blue) compared to ambient waters (dark blue). Open circles indicate individual neuston tow samples in slicks (*n* = 80) and ambient waters (*n* = 54).

Comparing fish families revealed that 88% (29/33) were at least twice as abundant in surface slicks as in ambient water (range 2.2–109.8 times, Fig. 5, Supplementary Table S4). These slick-associated taxa originate from a diversity of natal habitats. 95% of coral reef fish families, 71.4% of epipelagic families, and 83.3% of deep-water families (e.g., mesopelagic, bathypelagic, and deep-demersal) were at least twice as abundant in surface slicks as in ambient water. Seven abundant families were found nearly exclusively in slicks (> 90% relative abundance), including three families of reef fishes: triggerfishes (Balistidae), blennies (Blenniidae), and filefishes (Monacanthidae), as well as four families of epipelagic fishes: flyingfishes (Exocoetidae), needlefishes (Belonidae), halfbeaks (Hemiramphidae), and driftfishes (Nomeidae). In addition, larvae of five rarer (≤ 10 individuals total) reef fish families were found exclusively in slicks (Antennariidae, Apogonidae, Holocentridae, Isonidae, and Tetraodontidae). Note that for comparisons of abundance, we only included families that were present in four or more neuston tows (see “Methods—Statistical analyses”).

For species-level comparisons, 81 fish taxa were consistently and reliably identified to species, 38 of which were common (i.e., present in at least 4 tows). Comparing species revealed that 92.1% had mean densities in slicks at least twice those in ambient water (range 2.0–190.0 times; Supplementary Table S4). Additionally, 61% of species were found almost exclusively in slicks (> 90% relative abundance), and 26.3% were found only in slicks (Supplementary Table S5). The three most diverse families accumulating in slicks were flyingfishes (11 species), jacks (10 species) and goatfishes (6 species) (Supplementary Table S4; see Supplementary Note 2).

Slick nurseries support early life stages of at least 22 fisheries-harvested species (Supplementary Table S4). For example, pelagic predators like mahi-mahi (Dolphinfishes, *Coryphaena* spp.), swordfish (*Xiphias gladius*), marlin and spearfish (Istiophoridae)^40^ are commercially important to long-line and sportfishing industries. Mackerel scad (*Decapterus macarellus*) are an important fishery throughout the Pacific as both food and baitfish, particularly in Hawai‘i where they are the majority of inshore commercial catch^41,42^. Larger jacks (*Caranx* spp.) are highly prized targets for shoreline recreational fisherfolk^43^. Estuarine fishes, including juvenile mullet (*Neomyxus leuciscus*), milkfish (*Chanos chanos*), and flagtails (*Kuhlia* spp.), are major components of aquaculture where they are trapped wild, grown, and harvested in traditional Hawaiian fishponds^42^.

**Figure 6:**
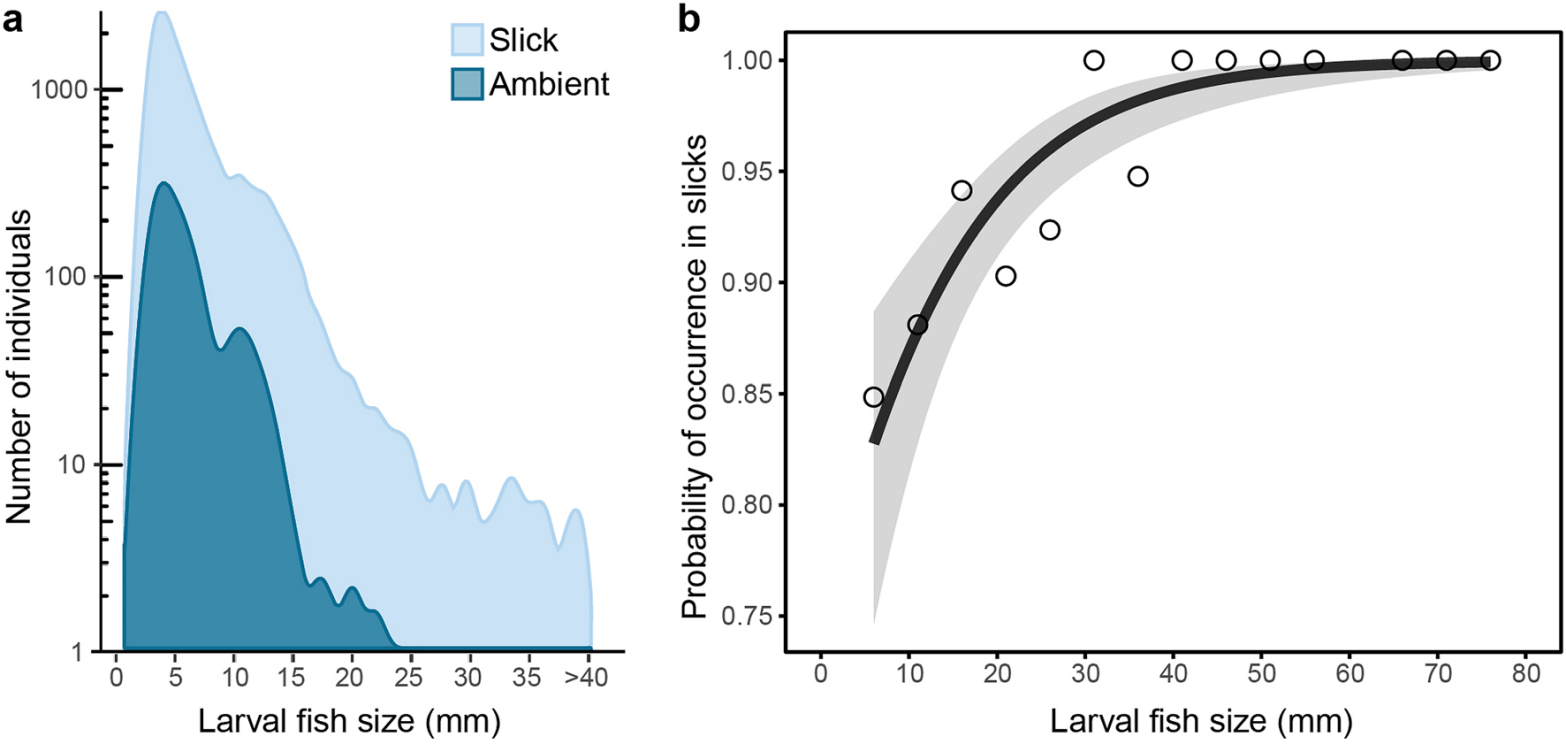
Larval fish size structure in slicks and ambient waters. (**a**) Size frequency histogram of larval total length (mm, in 1-mm size bins) in slicks (*n* = 11,772) and ambient waters (*n* = 1427). (**b**) Beta regression analysis of the predicted probability of larval fish occurrence in surface slicks versus ambient water as a function of larval fish size (total length, mm, in 5-mm size bins). Observed values are in open circles, black line is the modeled probability ± standard error (grey shading).

We found substantially greater fish abundance in slicks for every early life stage, from eggs to pelagic juveniles. Mean densities of fish eggs were 3.1 times higher in slicks (8.85 m^−3^ slicks; 2.85 m^−3^ ambient; P($\bar{d}$_*slick*_>$\bar{d}$_*ambient*_) ≥ 0.9999). Mean densities of every size class from preflexion hatchlings (2 mm total length, TL) through pelagic juveniles were at least 5.0 times as abundant in slicks than in ambient water. Size distributions were skewed, with orders of magnitude higher abundances of smaller larvae and relatively fewer later stages (Fig. 6). The size distribution is notably truncated in ambient waters with few individuals > 20 mm TL. Mean fish length was 14.6% larger in slicks (6.11 mm TL) compared to ambient (5.33 mm TL) (P($\bar{d}$_*slick*_>$\bar{d}$_*ambient*_) ≥ 0.9999, Fig. 6a). The overwhelming majority (91.6%) of fishes ≥ 10 mm (i.e., late-larvae and juveniles), were collected inside slicks. The probability of occurrence in slicks increased with fish size (beta regression: *Z* = 4.64, pseudo-*R*^2^ = 0.66, *P* < 0.000001, Fig. 6b). Therefore, size was a strong predictor of the probability of occurrence in slicks with late-larval and pelagic juvenile stages occurring almost exclusively in slicks. This pattern is consistent in most fish families we observed as demonstrated in family-specific size-frequency distributions (Supplementary Fig. S3).

Accumulation of debris could be an important factor driving larval fish behavioural attraction to slick habitats. The mass of floating debris was a significant predictor of larval fish density (*F*_*1,130*_ = 35.75, *P* < 0.0001), and in slicks the strength of this linear relationship increased with fish size class from *R*^2^ = 0.1 for larval fishes < 5 mm (*P* < 0.01), up to *R*^2^ = 0.46 for larval fishes > 20 mm (*P* < 0.01; Supplementary Fig. S4; Supplementary Note 3). In ambient water, the relationship between larval fish density and mass of debris was weaker (*R*^2^ < 0.18) and not significant for all size classes (*P* > 0.30). These results indicate that: (i) the positive association between larval fish density and floating debris increases with fish size class, (ii) this relationship is stronger in slicks than in ambient water, and (iii) debris accumulation in slicks may be a strong driver for attracting late-larval and juvenile fishes in these habitats.

The majority of slicks we surveyed in West Hawai‘i were classified as internal wave slicks (65%), followed by those generated by groundwater discharge (11.25%), tidal fronts (6.25%), headland fronts (2.5%), and unclassified (15%). The mechanism underlying slick formation was determined based on a variety of information including field observations, satellite images, tidal phase, and wind conditions (see “Methods”—“Classifying physical mechanisms”). In general, slick mechanisms can be distinguished by the following characteristics: internal wave slicks are parallel bands that travel in an onshore direction with consecutive propagating internal waves; groundwater discharge slicks are formed by fronts at the edge of a groundwater discharge plume that is colder and less salty than the ambient water; tidal front slicks form between deeper waters where open-water stratification dominates and shallower waters mixed by tidal currents interacting with the bottom; headland front slicks form between waters moving with the alongshore current and waters potentially “shadowed” from the alongshore current by the headland (see Supplementary Fig. S1).

For a regional perspective, we used satellite images to map the spatial coverage of surface slicks across a ~1000 km^2^ region of the ocean off West Hawai‘i (Fig. 1a, Supplementary Fig. S5) and then scaled our *in-situ* surveys (Fig. 1b) up to this region (see “Methods”—“Remote sensing” and Gove & Whitney et al.^34^). We estimate that surface slicks occupy 8.3 ± 2.4% (mean ± SD) of all nearshore (≤ 6.5 km) ocean surface habitat^34^ but contain 21.9 ± 2.4% of all neustonic larval invertebrates, 26.5 ± 2.8% of surface-dwelling zooplankton, and 39.1 ± 3.5% of neustonic larval fish (see “Methods”—“Scaling estimates”). We found that the estimated percentage that occurs in the top 1 m of slicks across this region increases with life stage, starting with fish eggs (22.9 ± 2.5%), and increasing from early larval stages < 10 mm (38.5 ± 3.5%) through late-larval stages ≥ 10 mm (46.3 ± 3.7%) then up to larger pelagic juveniles ≥ 30 mm (90.9 ± 1.3%), which were almost exclusively found in slicks. We also estimate that slicks contained 75.4 ± 2.9% of floating organic debris and 95.7 ± 0.6% of floating plastic debris, which combined represents 86.4 ± 1.9% of all floating debris in our study area.

## Discussion

**Figure 7:**
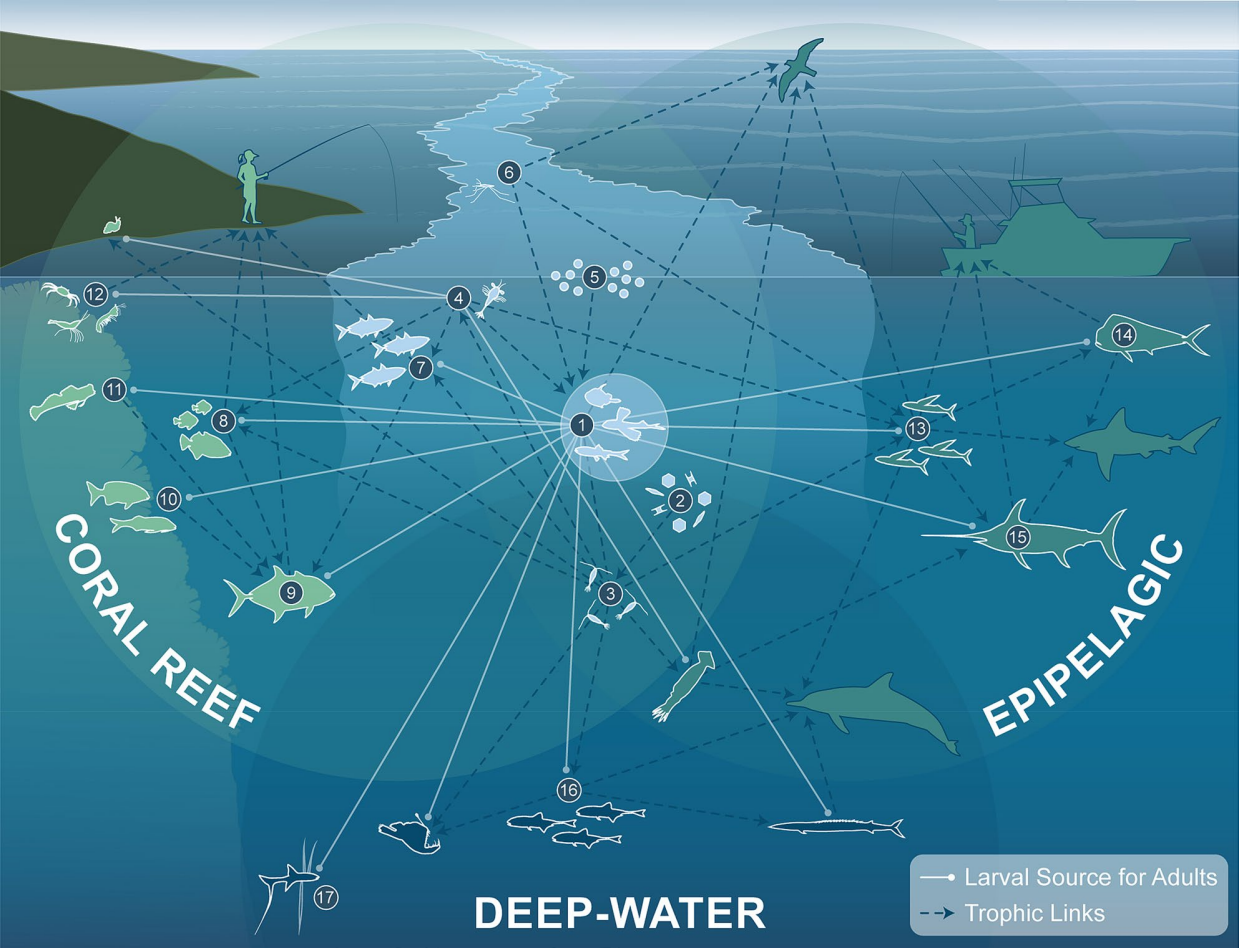
Conceptual diagram illustrating key examples of the ecological connections and functions enhanced by surface slick nurseries. (1) Larval and juvenile stages of fishes from many ocean habitats aggregate in slicks in order to capitalize on dense concentrations of prey (2, phytoplankton, 3, zooplankton, 4, larval invertebrates, 5, eggs, and 6, insects). The increased predator–prey overlap in slicks increases energy flow that propagates up the food-web (dotted blue lines show trophic links), enhancing energy available to higher trophic level predators (icons outlined in blue) including humans. More than 100 species of fishes develop and grow in surface slick nurseries before transitioning to adults (solid white lines radiating outward) in Coral Reefs (7–12), Epipelagic (13–15), and Deep-water (16–17) ocean habitats. As adults these taxa (icons outlined in white) play important ecological functions and provide fisheries resources to local human populations. For example, coastal schooling fishes (7, mackerel scad) are important food and bait fish for humans. Planktivorous fish (8, some damselfishes and triggerfishes) transfer energy from zooplankton up to reef predators^47^ like jacks (9), which provide top-down control of reefs^48^ and are important targets for shoreline recreational fisherfolk^43^. Grazers (10, chubs) help keep coral reefs from being overgrown by macroalgae^49^. Cryptobenthic fishes such as blennies (11) and benthic macrocrustaceans (12, shrimp, stomatopods, crabs) comprise most of the consumed biomass on reefs^50,51^. In the pelagic ocean, flyingfishes (13) channel energy and nutrients from zooplankton to pelagic predators such as mahi-mahi (14) and billfish (15), both of which utilize slicks as nursery habitat. Larvae of mesopelagic fishes like lanternfish (16) and bathydemersal tripod fishes (17) utilize these surface hotspots before descending to deep-water adult habitat. Surface slicks are clearly the focal point for numerous trophic and larval connections that are foundational for marine ecosystem function. For clarity, the diagram only illustrates several key examples and does not represent the full breadth of ecological connections enhanced by surface slicks.

We provide ecosystem-scale evidence that surface slicks are nursery habitat for a diverse assemblage of neustonic larval fishes and invertebrates in the ocean. Our findings suggest that by enhancing trophic connections and larval supply, slicks have far-reaching impacts on coastal ecosystems and the fisheries they support (Fig. 7). While larvae of most fish species are distributed throughout the upper 100 m of the ocean^44,45^, slicks provide habitat for neustonic larvae of at least 112 species of ecologically and commercially important fishes representing ~  9% of the 1200 + fish species recorded in the Hawaiian Archipelago^36,46^. These nurseries host early life stages of fishes that originate from a variety of natal habitats spanning shallow coral reefs (e.g., triggerfishes and jacks), out into the open ocean (e.g., flyingfishes and mahi-mahi), down into the mesopelagic (e.g., lanternfishes and bristlemouths), and even to the bottom of the deep-sea (e.g., tripodfishes) (Fig. 7). By providing survival advantages, slick nurseries likely contribute disproportionately to the replenishment of adult populations in these diverse marine habitats.

The growth and survival of developing larvae are largely dependent on the availability of patchily distributed prey resources^52,53^. Slicks cover ~ 8% of the 1000 km^2^ study area as estimated from remote sensing analysis^34^, yet they make disproportionately large contributions to the distribution of neustonic larvae and zooplanktonic prey. More than 39% and 26% of neustonic fish larvae and surface-oriented zooplankton, respectively, were condensed into these narrow convergence zones, making slicks reliably productive foraging grounds for larvae. Moreover, slicks contain a diverse assemblage of prey, including copepods, the primary prey item of most marine fish larvae^54–56^, as well as gastropod veligers, terrestrial insects, water striders, and a variety of microcrustaceans (Fig. 3). A higher diversity of prey types and sizes supports a more varied assemblage of larval fish predators^55,57^, and can enhance trophic transfer efficiency^58^. The enhanced spatial overlap between larval fish and diverse prey groups (Supplementary Fig. S2) supports the notion of intensification of trophic interactions in these enrichment zones. Predators and prey are highly aggregated in both horizontal and vertical dimensions. Similar to thin layers^59–61^, these aggregations create spatially-explicit foraging hotspots with increased probability of prey encounters for larval fish predators, which may translate to increased foraging success, faster growth^62^, and higher survivorship^53^.

We estimate that slicks contain 75.4% of all floating organic debris in our ~ 1000 km^2^ study area and therefore offer a selective advantage for vulnerable early life stages seeking shelter in the mostly structureless pelagic environment. Floating debris in the ocean provides refuge from predation via either direct shelter or obscuring the view of predators^63,64^. This predator refuge benefit may, in part, be offset by potentially higher predation risks if slick convergence zones also attract more predators, which has been observed in some frontal systems (e.g., riverine plumes^52^) and at the pycnocline (e.g., thin layers^60,61^), or by exposure to hazardous plastic debris^34^. Slicks contained 95.7% of all plastic debris (by weight) and 91.8% (by density^34^) encountered in our study area. Although some plastic may provide shelter from predators, chronic exposure to plastics could adversely affect animal feeding, growth, and survival^65^. Furthermore, smaller prey-size microplastics are highly concentrated in slicks and are being ingested by larval fish at higher rates than in ambient water^34^. The deleterious impacts of plastic ingestion to marine larval fishes are not well understood. But studies of adults reveal several adverse physiological effects including malnutrition^66^, gut blockage and perforation^67^, and decreased predator avoidance^68^. The antagonism between the advantageous effects of organic debris and the negative effects of plastic debris in these habitats is not yet resolved. Nevertheless, the concentration of hazardous plastic debris in slicks certainly represents a threat to the biodiversity hosted in these nurseries and the human communities they enhance^34^.

Phytoplankton, zooplankton, fish, and debris are concentrated in slicks by a combination of convergent circulation^69^ and behavioural feedback^22,26,70^. Convergent flow accumulates passive, buoyant particles, such as floating debris^22,71^, as well as semi-mobile surface-orienting zooplankton and small larvae^26,72,73^. Subsequently, aggregations of prey and debris attract larger, more mobile larval and juvenile fish^22,40,63,64^ that have strong swimming and sensory abilities^74^. Swimming competency increases with larval fish size^1^, with larger larvae (> 10 mm) being capable of swimming horizontally against strong currents for extended durations^74^. We found that the probability of larval occurrence in slicks increases with fish size, suggesting that competent swimmers are actively targeting surface slicks to capitalize on concentrated prey resources and shelter from predators.

We checked for confounding effects in our field sampling that might impact findings. We had sampled during 29 days over three years using two types of gear. However, sampling year, day, gear type and sampling order had no significant effect on response ratios of organisms in slicks versus ambient water (Supplementary Note 3). We conclude that these effects explain little of the variation in response ratios and therefore have limited impact. With any plankton survey, net avoidance behavior is a potential source of error, particularly for larger fish larvae with strong swimming abilities. Specifically, the flight response by larval fishes in the presence of floating debris could be different (larvae may seek shelter around debris as the net approaches) than in the absence of debris (larvae may swim downward to avoid capture). Because debris is disproportionately accumulated in slicks, this potential difference in behavior could result in selectively under-sampling larger individuals in ambient water. Although we cannot completely rule out this possibility, evidence suggests that net avoidance is unlikely to be a confounding factor in our results. First, we demonstrated that habitat (slick:ambient) was a significant predictor of larval fish density, even after controlling for the amount of debris. Second, we did not find a relationship between response ratio of larval fishes and time of day, which would otherwise indicate a light-dependent pattern driven by net avoidance. Third, the strong association of larger larvae with debris in slicks (Supplementary Fig. S4) strongly suggests a behavioral attraction to debris in slick habitats as opposed to net avoidance in ambient water.

Surface slicks may also drive larval transport and influence larval retention nearshore. Previous studies have documented the onshore transport of larval invertebrates and fishes in internal waves slicks^8,22,24,26^, suggesting a positive link between the prevalence of surface slicks and larval settlement^23^. For coral reef-associated species, shoreward progression presumably aids in keeping larval reef fish in the vicinity of reefs and supports the delivery of competent larvae to settlement habitat^23,75^. Therefore, by either preventing advection offshore or transporting pre-settlement larvae onshore, aggregation in slicks is likely to reduce dispersal distances and enhance self-recruitment^75,76^ for coral reef animals. Such mechanisms that facilitate local retention are likely extremely important to the maintenance of island-reef ecosystems^2^. However, the perceived benefits of shoreward transport are less clear for epipelagic and deep-water species, as coral reef ecosystems represent unsuitable settlement habitat for these organisms. Reef noise may act as a deterrent cue for non-reef species^77^, but the sensory cues and behavioural adjustments that epipelagic and deep-water species employ to avoid shorelines are largely unknown.

Surface slicks have profound impacts on larval distribution of multiple forage species that play critical roles in pelagic food webs. Flyingfishes, halfbeaks, needlefishes, silversides, and mackerel scad are important forage species to higher trophic level predators and were all strongly concentrated in slicks (Fig. 5; Supplemental Note 2). Such species channel energy and nutrients from plankton to top predators and thus play an important role in regulating ecosystem dynamics^78^. For example, flyingfishes, which were nearly exclusively found in slicks, are a significant component of the diet of multiple predators, including tuna, billfish, and mahi-mahi^79^, as well as 95% of all Hawaiian seabirds^80^. The importance of slicks to the early development of flyingfishes and other forage species clearly has far-reaching implications to the pelagic food web (Fig. 7). Increases in prey abundance provide more energy flow to commercially important species and enhance productivity back to land via trophic links with seabirds^81^ and humans^82^.

Biophysical interactions with surface slicks drive changes in pelagic community structure that translate to higher abundance, greater diversity, and increased trophic interactions. This structuring is expected to influence trophic transfer by aggregating predators and prey in spatially explicit hotspots where encounter rates are enhanced^83^. Increased predator–prey overlap in slicks (similar to ocean fronts^16^) amplifies the flow of energy up the food-web (Fig. 7) and ultimately enhances ecosystem and fisheries productivity^16,83^. Although individual slicks are localized and ephemeral, the continual formation of many slicks have a synergistic effect. Woodson^84^ estimated that if tidally generated internal waves increase encounter rates by a factor of ten^13^, it could lead to a 75% overall increase in coastal ecosystem productivity. Because internal wave slicks are a dominant and regular surface feature in this region^37^, enhancements of prey consumption could play a major role in enriching ecosystem productivity^16,83,84^.

Surface slicks are dynamic and variable ocean features. Ephemerality is on the order of hours for internal wave slicks, driven in part by tidal phase and background stratification^19^. In contrast, the pelagic larval duration of many tropical marine species is on the order of weeks to months^85^. To realize the benefits from the accumulation of food resources, larvae would have to utilize multiple slicks over the course of their larval duration. However, while slicks are temporally variable, they are also prevalent, broadly dispersed across much of the region, and typically separated by just a few hundred meters (Fig. 1a ^34^). Given that competent swimming individuals are capable of traveling tens of kilometers^86^, we postulate that larval fishes, particularly larger, more-well developed individuals, are targeting and transiting between slicks to capitalize on increased prey availability and shelter throughout their larval phase.

We show that biophysical interactions with surface slicks structure the surface ocean and strongly influence the distribution and ecology of neustonic larvae and zooplankton. Surface slicks provide critical early life habitat for fish development, from eggs to larvae to juveniles, with some neustonic taxa depending strongly on slicks for growth and transport to adult habitat. Slicks concentrate food and shelter for developing larvae, potentially increasing survival rates and bolstering recruitment of young fish and invertebrates, which are important to humans for fisheries, recreation, and other ecosystem services. By providing these survival advantages, surface slicks enhance larval supply and thus replenishment of adult populations in coral reef, epipelagic, and deep-water ecosystems. In recruitment-limited systems like coral reefs, the number of recruiting juveniles determines the productivity of the system and its resilience to disturbance^3,4^. The communities affected by slicks, via increased larval supply and energy flow, should therefore experience enhanced ecological resilience due to increased replenishment and productivity^87,88^. Currently these enhancements to productivity are not accounted for in ecosystem or fisheries models. Taken together, our work suggests that surface slicks play a previously underappreciated but critically important role in larval ecology, fish replenishment, and ecosystem functioning in tropical and subtropical systems.

## Methods

### Study area

This study took place in the coastal waters of leeward Hawai‘i Island, which is located at the southeastern end of the Hawaiian archipelago (Fig. 1). The ship-based survey area encompassed coastal waters within 6.5 km of western Hawai‘i Island spanning ~ 100 km from Keawanui Bay (20.11° N, −155.90° W) south to Miloli‘i (19.23° N, −155.92° W; Fig. 1). Surveys were conducted during three separate field expeditions in September 2016 (21 days), April 2017 (14 days), and July 2018 (12 days). We selected sites in waters off the entire West Hawai‘i coastline to represent a range of environments. Furthermore, we conducted surveys between the months of April and September to coincide with the peak spawning and recruitment period for the majority of Hawaiian coral reef fishes^89^.

Hawai‘i Island is typical of high volcanic islands that are located throughout much of the tropical and subtropical Pacific. Shallow depths of West Hawai‘i are dominated by essentially continuous coral reefs along the coastline. The island has a complex bathymetry with contrasting features both across and along the coastal waters of the island. In the north, a broad shelf with gradually sloping bathymetry extends tens of kilometres from shore, whereas in the south the bathymetry drops dramatically to depths of 1000 m within 2000 m or less of the shoreline. In the south, there is gradual sloping bathymetry close to shore (within 100 s of meters) that transitions to an abrupt shelf break and steeply sloping bathymetry further offshore, which are important for internal wave generation^33,90^. The shoreward propagation of internal waves is affected by bathymetric slope, and this region is characterized as having highly active internal wave generation^33^. While internal waves and associated slicks are a common coastal feature around the world^17^, they are particularly prominent in tropical nearshore systems like the coastal waters off West Hawai‘i (Fig. 1, Supplementary Fig. S5^34^). The conditions that lead to the formation of tidally generated internal waves include density stratification and sharp bathymetric changes^84^. In the lee of islands in the tropics, low winds allow for stable stratification, and thus internal wave generation, along with sufficiently calm waters for slick formation throughout the year.

The Hawaiian Archipelago supports a high diversity of marine fishes, with at least 1200 recorded species, including more than 500 shorefishes, many of which inhabit coral reefs^36,46^. As a consequence of the complex island bathymetry, multiple marine habitats are compressed in horizontal distance from shore, and early life stages from diverse communities of coral reef, epipelagic, and deep-water (i.e., mesopelagic, bathypelagic, and deep-living demersal) fishes overlap in close proximity to islands^35,36^. The high biodiversity, complex geography, and high frequency of slicks make this region an ideal model system to evaluate the ecological effects of slicks on tropical marine ecosystems.

### Neuston tows

We examined abundances of fish, zooplankton, and debris in slicks and in corresponding samples outside of slicks, hereafter referred to as ambient waters. Each plankton transect consisted of a single ~ 500 m neuston tow. Transects were performed both inside surface slicks and in nearby ambient waters. Tows in ambient waters were typically run parallel with and between slicks, at a distance of 200–500 m.

Surface (≤ 1 m depth) planktonic organisms were sampled by towing a straight-conical ring-net (1 m diameter, 4.5 m length, 335 μm mesh; Sea-Gear Corporation) behind a small boat. Surface tows were conducted using a custom-built tow design similar to a Manta net^91^. The tow set-up sampled the air–water interface to ~ 1 m depth. The net was lashed to an aluminium square frame (40 mm diameter) fitted with surface displacement floats to keep the top of the net at the air–water interface. The net was towed using an asymmetrical bridle and paravane (1.27 cm starboard) to ensure the net frame was clear of the towing vessel’s wake. The surface net was fitted with a 300 μm mesh soft cod end. A flowmeter (Sea-Gear Corporation) was mounted in the mouth of the net (mouth area = 0.79 m^2^), providing the total volume sampled for each tow. Surface slick and ambient water neuston tows were conducted for ~ 8 min at a speed of ~ 4 km h^−1^. Transect location and length were measured using a hand-held GPS (GPSMAP78; Garmin). Tow length was 495 ± 157 m (mean ± s.d., Supplementary Table S6). At times, truncation of slicks or loss of visual cues resulted in shorter tow lengths. All transects were conducted from 150–6500 m horizontal distance from shore (mean ± s.d.: 1861 ± 1605 m; Supplementary Table S6) over bottom depths of 12–1689 m (mean ± s.d.: 412 ± 460 m).

In 2017, 16 neuston tows were conducted from the NOAA Ship *Oscar Elton Sette* using a 1.8 m (6 ft.) Isaacs-Kidd (IK) trawl^92^ equipped with a winged depressor and a 505 μm mesh net. The IK was mounted from a J-frame crane along the midship cut-out, sampled alongside to mitigate disturbance from the ship, and fished as a neuston net, sampling from slightly above the air-sea interface down to ~ 1.5 m depth. The IK was fitted with a flowmeter (Sea-Gear Corporation) mounted in the mouth of the net (mouth area = 2.75 m^2^) providing the approximate volume sampled for each tow. Neuston tows were conducted for ~ 12 min at a speed of ~ 6 km h^−1^. Transect location and length were measured using a hand-held GPS (GPSMAP78; Garmin). Tow lengths for IK neuston tows conducted from the ship were 1012 ± 356 m (mean ± s.d.; Supplementary Table S6).

Surface slicks were identified and sampled based on visual assessment. Slicks were determined and selected by locating smooth waters with clearly identifiable edges meeting rippled water that were separated by 5–200 m in width and extended at least 500 m. Generally, slicks were only visible at wind speeds between 4 and 20 km h^−1^. At winds < 4 km h^−1^ the ocean surface was predominantly smooth, while at winds > 20 km h^−1^ the ocean surface was predominantly rippled. In either case, slicks were indiscernible and therefore could not be sampled. Transects within slicks were conducted using a sinuous tow pattern enabling the centre and edges to be sampled. Plankton samples were secured and preserved immediately in the field in 95% undenatured ethanol and then transferred to jars with fresh ethanol upon returning to the lab. The plankton net was cleaned between transects by removing the cod end, inverting the net, and hand scrubbing the net to remove residual plankton and algae. Nearby ambient waters were sampled on average 774 m (SD = 581 m, range 98–3326 m) away from each sampled slick. In total, we had *n* = 80 tows from surface slicks and *n* = 54 from ambient waters (Fig. 1, Supplementary Table S6). Our sampling design was to pair each slick sampled with an ambient sample. However, because of inclement weather, changing wind conditions, mechanical failures, and other operational constraints, we were unable to achieve our sampling design for all slicks sampled. We ultimately had 52 samples from surface slicks that were paired with ambient waters.

The time between neuston tows was typically > 30 min (median = 33, mean = 62, SD = 64 min). Thus, we consider the separation in both space (> 100 m) and time (> 30 min) between tows in slicks and ambient water to mitigate the potential for boat disturbance (i.e., propeller noise and wake) on catches between habitats. Although there was a bias toward sampling slick habitats first (in 41 of 52 paired transects), we found that sampling order did not have a significant effect on log response ratios (densities of fish in slicks compared to ambient water, see “Methods”—Statistical analyses below) for all fish larvae (ANOVA; *F*_1,50_ = 0.029; *P* = 0.86) or larger fishes (TL > 20 mm, *F*_1,49_ = 0.32; *P* = 0.57). Therefore, we conclude that sampling order does not represent a confounding factor on our results.

### Classifying slick physical mechanisms

In this study, the teams initially set out to study internal wave slicks and were specifically looking for slicks parallel to bathymetry, spaced 50–200 m apart, and propagating onshore at relatively low speeds. A number of slicks were sampled that were later classified as being generated by other mechanisms (i.e., tidal fronts, headland fronts, or groundwater discharge). The analysis for this contribution groups all slick data together, regardless of generating mechanism. Mechanisms were inferred based on field observations, GPS mapping, satellite images, tidal phase, wind conditions, and maps of submarine groundwater discharge (SGD)^37^. In general, parallel lines of slicks propagating towards shore at the appropriate spacing and velocity were likely internal wave slicks; stationary slicks close to shore and near known SGD plumes were likely SGD fronts; stationary slicks close to and parallel to the shore were likely tidal fronts; and stationary slicks located near headlands were likely headland fronts. Langmuir cells can also generate rows of slicks (windrows) parallel to wind direction^25^; however, we did not sample any Langmuir cell slicks in this study. Please see Smith et al.^37^ for a detailed analysis of the underlying physical mechanisms that generate nearshore slicks and the variability of accumulation by different drivers. See Supplementary Fig. S1 for illustrations of the flows associated with mechanisms^93,94^.

### Sample processing

Following the cruises, preserved samples containing organisms and debris were identified under dissecting microscopes (2–30× magnification) and manually sorted into key groups: invertebrates, fish larvae, fish eggs, and debris (organic and synthetic). All fish larvae were identified to the lowest taxonomic level possible (operational taxonomic unit) using morphological characters, measured to total length (TL, to nearest mm), and counted for each sample in its entirety (i.e., no subsamples were taken). Larval fish identification relied upon a number of sources^95–100^. We follow terminology used by Leis et al.^99,101^ in the use of a broad, ecological definition of larvae to include all post-hatching pelagic life-history stages. For coral reef associated taxa this is equivalent to the pre-settlement stage (sensu Kingsford and Milicich^102^), and is inclusive of both larvae and pelagic juveniles. There is no generally accepted definition for when a larva becomes a juvenile, and for most pelagic taxa this transition is not well resolved^99,101^. Therefore, our analyses do not make the distinction between larval and juvenile stages, but generally we refer to our samples as containing both larvae and juveniles. The remaining bulk zooplankton samples were size-fractioned into three fractions: 0.3–1.0 mm, 1.0–2.0 mm, and > 2 mm, sub-sampled using a Folsom plankton splitter, enumerated, and identified into broad taxonomic groups and life stages when possible. The marine water striders (*Halobates* spp.) were identified to species, life stage, and sex using characteristics outlined in Anderson and Cheng^103^ and enumerated. All counts were standardized to the volume of water sampled for each tow and converted to densities (individuals m^–3^ or individuals 1000 m^−3^, where noted). For each fish taxa, we used habitat classifications from FishBase^104^ to assign adult habitat into three broad habitat groups: (1) Coral reef includes all reef-associated shorefishes, (2) Epipelagic, and (3) Deep-water, which includes mesopelagic, bathypelagic, rariphotic, bathyal, abyssal, and deep-living demersal fishes. For families that have members in multiple habitat types, we used a majority rule.

### DNA barcoding

Several families have poorly resolved taxonomic resources to identify larvae to genus or species. To improve genus and species-level resolution of difficult to identify families, we sequenced a total of 133 individuals from four common fish families with poor taxonomic resources, including jacks (Carangidae), triggerfishes (Balistidae), flyingfishes (Exocoetidae), and goatfishes (Mullidae). We selected a variety of individuals for each target group that represented different larval types and across as much of the available size distribution as possible. We took photographs and detailed notes of each individual specimen sequenced to serve as vouchers. All tissues were sent to the Canadian Centre for DNA Barcoding (CCDB, University of Guelph) for molecular analysis. For each extract, DNA barcodes were generated for the 5′ region of the mitochondrial COI gene following the CCDB protocols for PCR and sequencing (detailed in Hajibabaei et al.^105^). Complete taxonomic information, collection records, voucher images, and sequences for 133 specimens are publicly available through The Barcode of Life Database (BOLD, http://www.boldsystems.org, Process IDs: SLICK001-19:SLICK190-19). We compared the sequences obtained from these specimens with reference sequences available in BOLD and Genbank. We used high confidence (> 98% similarity) identifications to group specimen images by genetically-identified genus and species. Images and detailed morphological notes were then used to re-examine specimens for diagnostic morphological traits. For all four families, we were able to use refined morphological criteria based on identities of barcoded individuals to identify other individuals at the genus and species level (when possible).

### Debris

Plastics were manually extracted from neuston samples under stereoscopic dissecting microscopes and identified visually by their colour, shape, and texture. We followed Norén^106^ and Hidalgo-Ruz, et al.^107^ for visual identification of synthetic particles and used the following criteria: (1) texture should be hard, durable and not easily broken or crushed when squeezed with forceps; (2) no cellular or organic structures should be visible; (3) colours should be homogenous; and (4) fibres should have uniform diameter throughout their entire length. Plastics manually extracted from each sample were dried overnight and weighed. To mitigate potential contamination of synthetic particles that could have been shed from the vessel and sampling gear, we excluded all paint chips and any fragments matching colours of neuston net float or paravane. Naturally occurring organic debris, including macroalgae, plant matter (e.g., leaves, twigs, seeds), and animal matter (e.g., feathers, arthropod molts) was manually extracted from neuston samples, dried overnight to allow all residual ethanol to evaporate, and then weighed. Dry weights of plastic and organic debris were standardized to volume of water sampled for each tow (mg m^−3^).

### Water samples

Water samples for chlorophyll fluorescence analyses were taken from the surface at the origin of the neuston tow. Samples for chlorophyll were collected by hand using a 250 ml dark Nalgene bottle and immediately placed on ice in the field. Water samples were filtered onto 25 mm glass microfiber filters (Whatman), placed in 10 ml of 90% acetone, frozen for 24 h, and then analysed for chlorophyll*-a* and total chlorophyll concentration (mg m^–3^) using a Turner Designs model 10AU field fluorometer. Water samples from inside slicks were always paired with samples taken in ambient waters.

### Remote sensing

Planet Dove satellite images (https://www.planet.com) were utilized due to their daily revisit frequency and high spatial resolution (3.7 m). Our mapping approach utilized the contrast between the surface texture of slicks and regular seawater, which is most significant when sun glint is observed in the satellite images. A total of 97 cloud-free, sun glint-saturated Dove reflectance images were selected from Planet to cover the study area. Images were selected in the following time steps in 2018 to assess surface slick spatial distribution and extent: August 31, September 23, October 3, and October 11 (Fig. 1a and Supplementary Fig. S5). The spatial extent of remote sensing detection was constrained to the spatial extent of our neuston plankton samples (≤ 6.5 km from shore) across the West Hawai‘i coastline. See Gove & Whitney et al.^34^ for further details on the identification of surface slicks from satellite imagery.

### Geospatial analysis

Bathymetry data (Fig. 1 and Supplementary Fig. S5) were obtained from the University of Hawai‘i (http://www.soest.hawaii.edu/HMRG/multibeam/bathymetry.php). All geospatial analyses were performed in ArcGIS Desktop 10.6 software (http://desktop.arcgis.com/) with the extensions and tools specified below. Geospatial information was derived for the surface slicks identified with Planet Dove satellite imagery (see “Methods”—“Remote sensing” and detailed description in Gove & Whitney et al.^34^). Distance to shore for each neuston transect (Supplementary Table S6) was calculated as the shortest distance from the centroid of the GPS track to the shoreline using Near Analysis tools.

### Scaling estimates

To estimate the percentage of larval fish and plastics in surface slicks across the ~ 1000 km^2^ study area, we first multiplied the ocean surface area of slicks and ambient waters, as estimated from the four remotely sensed time points (see “Methods”—“Remote sensing”), by each of the 10,000 bootstrap replicates of mean larval fish and plastic densities (see Gove & Whitney et al.^34^ for detailed methods). We then calculated the mean of these 10,000 population estimates for each group in slicks as a percentage of the total study area observed for each time point. All calculations were constrained to the spatial extent of our neuston tows (≤ 6.5 km).

### Statistical analyses

Individual neuston tow density values were calculated by dividing the numerical abundance of each taxon or group by the total volume of water sampled for each tow. Non-parametric bootstrap was used to explicitly investigate the uncertainty (i.e., 95% bootstrap intervals) associated with mean density values in each group. The bootstrap was based on random sampling (with replacement) from the original densities for each group separately. Non-parametric bootstrap was preferred to avoid explicit assumptions about the distribution of density values. The bootstrap intervals for mean densities were based on 10,000 bootstrap replicates. The same approach was applied to larval fish size, except with 20,000 bootstrap replicates owing to the large sample size (*n* = 13,217).

A permutation test was used to calculate the empirical probability that the mean density ($\bar{d}$) of chlorophyll, zooplankton, and larval fish (including individual taxa) inside slicks ($\bar{d}$_*slick*_) is larger than mean density in ambient waters ($\bar{d}$_*ambient*_) in our study:

$$\mathrm{P}\left(\left({\bar{d}}_{\text{slick}}>{\bar{d}}_{\text{ambient}}\right) \ge \left(1-\frac{1}{\text{nboot}}\right)\right),$$

where *nboot* is the number of bootstrap replicates. The empirical probability was calculated by randomly permuting the group labels (slick and ambient), each time recalculating the difference between mean group densities. P($\bar{d}$_*slick*_>$\bar{d}$_*ambient*_) was then calculated as the proportion of replicates for which the permuted difference of means was larger than the difference of group means in the original data.

We quantified how taxon density was influenced by surface slicks using the response ratio (e.g., Hedges et al.^108^), which measures the magnitude in effect of two treatments and typically refers to the ratio of mean outcome of an experimental group to that in a control group. Here we define response ratio as the ratio of mean taxon density in slicks to ambient water [$\bar{d}$_*slick(i)*_/$\bar{d}$_*ambient(i)*_]. We calculated this ratio for each of the 10,000 bootstrap replicates (_i_) to produce a distribution from which we could present the mean and 95% bootstrap intervals. To correct for instances where mean estimates in ambient habitats ($\bar{d}$_*ambient(i)*_) equal zero (i.e., denominator equal zero and results in undefined ratios), we divided the $\bar{d}$_*slick*_ (in each bootstrap replicate) by the mean $\bar{d}$_*ambient*_ of all 10,000 replicates. We acknowledge that the uncertainty in the ratio bootstrap intervals will be underestimated as we are not completely accounting for the variability in the mean replicates; however, we considered this a reasonable compromise to enable comparable estimates of bootstrap intervals for a wide diversity of groups. For statistical analyses, we excluded 21 of 54 fish families we considered too rare to classify (i.e., present in fewer than 4 tows). We define slick-associated taxa as those taxa with mean densities in slicks at least twice that in ambient water (Ratio Slick:Ambient ≥ 2) or taxa only recorded in slicks.

To compare the diversity and richness of larval fish communities in slicks versus ambient water, we used the Shannon-Weaner index (H′), and species richness (S) using the *vegan* package v2.2 in R^109^ and tested for differences in means using the bootstrap permutations procedure described above.

In order to examine how slicks influence the ichthyoplankton community structure, we used a distance-based redundancy analysis (dbRDA^110^). The response matrix consisted of Bray–Curtis distances (using log-transformed abundance) of 33 larval fish families (present in ≥ 4 tows) per transect, and surface state (slick vs. ambient) was isolated as the only constraining variable. The dbRDA model was conditional on the site to control for site-specific effects not focal to our questions^111^. The dbRDA was conducted using the *vegan* package v2.2^109^ in R. Significance was tested by permutation (100,000 steps), and results were visualized with biplots using linear constraint scores for the first two RDA axes. Because only one constrained axis is obtained, the ordination graph uses the first residual axis as the second (y) axis. All densities were log transformed using the decostand function in *vegan*^109^.

To explore how slicks influenced the co-occurrence of larval fish and their prey, we calculated Spearman’s correlation coefficients using log-transformed densities (individuals m^-3^ per tow) of larval fish and zooplankton prey groups in both ambient water (*n* = 54 tows) and in surface slicks (*n* = 84 tows). Spearman's correlations and *P*-values were calculated using *Hmisc*^112^ and visualized using *corrplot*^113^ in R.

We used beta regression to explore the relationship between the probability of occurrence in slicks and larval fish size (total length). The relative abundance (proportion found in slicks compared to ambient) was calculated for each size class (5 mm increment bins) using bootstrapped mean densities in slicks divided by the sum of mean densities in slicks and in ambient waters. Since the predicted variable (relative abundance) is a rate ranging from 0 to 1, we used a beta regression model with the *betareg*^114^ package. We explored the linear relationships between fish density and floating debris as a function of fish size and habitat. Using independent one-way ANOVAs, we tested the effect of log-transformed debris mass (mg m^−3^, organic and plastic) on log-transformed fish density (individuals m^−3^; excluding zero densities) for five size-classes of larval fishes in 5-mm bins (< 5 mm, 6–10 mm, 11–15 mm, 16–20 mm, > 20 mm) in each habitat type (slicks and ambient water). Using an ANOVA, we tested for effects of sampling year, day, gear type (1 m vs. 6 ft Isaacs-Kidd trawl), and sampling order (slick or ambient tows first) on log-transformed response ratios of four major functional groups (total fish, juvenile fish, zooplankton, and total debris) calculated from 52 paired tow samples. To address the potential issue of net avoidance behavior we explored whether response ratios varied with respect to time of day. We follow the assumption that if net avoidance is a significant issue, we would expect to find a pattern between response ratios and time of day, with the greatest probability of net avoidance during solar noon when light levels and visibility are highest. We intentionally did not sample near sunrise, sunset or at night, and therefore cannot explore this relationship using those extremes of the lighting continuum, but can use the range of times we sampled (0800–1600). To test the potential confounding impact of light levels, we performed a one-way ANOVA using time of day (minutes from solar noon) as predictor of log response ratios of total fish density and juvenile fish density (TL > 20 mm) using the 52 paired tow samples. To explore the interaction of debris and light, we performed an ANCOVA with debris mass (mg m^−3^, log transformed) and time (minutes from solar noon) as covariates, habitat (slick vs. ambient) as the factor, and fish density (log transformed, excluding zero densities) as the response variable using all 134 tows (n = 80 slicks, n = 54 ambient tows). We conducted three independent ANCOVA tests with varying fish datasets including all larval fishes, small fishes (TL < 10 mm), and large fishes (TL > 10 mm). All data analyses were performed in R v3.4.0^115^.

### Reporting summary

All methods were carried out in accordance with relevant guidelines and regulations and all work was done under the guidance and approval of the National Oceanic and Atmospheric Administration. The study used environmental samples of zooplankton including ichthyoplankton, which were preserved immediately upon collection in 95% ethanol, and no experiments were conducted with live vertebrates.

## Supplementary Information

Supplementary Tables.

Supplementary Information.

## Data Availability

Data supporting the findings of this study are available from GitHub (https://github.com/jonwhit/SlickNurseries). New sequence data generated for this study were deposited in The Barcode of Life Database (BOLD, http://www.boldsystems.org, Process IDs: SLICK001-19:SLICK190-19).

## Code availability

The R script for bootstrapping procedure used in this paper is available from GitHub (https://github.com/jonwhit/SlickNurseries).
