# Supplementary material for: Surface slicks are pelagic nurseries for diverse ocean fauna
Source: Sci Rep. 2021 Feb 4;11:3197. doi: 10.1038/s41598-021-81407-0 (PMC7862242; doi:10.1038/s41598-021-81407-0)
Supplement: Supplementary file 2 — Supplementary Information. [file 41598_2021_81407_MOESM2_ESM.pdf]

# Supplementary Information

for

# "Surface slicks are pelagic nurseries for diverse ocean fauna"

Jonathan L. Whitney[1,2,3*], Jamison M. Gove[2], Margaret A. McManus[3], Katharine A. Smith[1,3], Joey Lecky[2,4], Philipp Neubauer[5], Jana E. Phipps[1,2], Emily A. Contreras[1,2], Donald R. Kobayashi[2], and Gregory P. Asner[6].

[1]Joint Institute for Marine and Atmospheric Research, University of Hawai'i at Mānoa, Honolulu, HI 96822, USA. [2]Pacific Islands Fisheries Science Center, National Oceanic and Atmospheric Administration, Honolulu, HI 96818, USA. [3]Department of Oceanography, University of Hawai'i at Mānoa, Honolulu, HI 96822, USA. [4]Lynker Technologies LLC, Marine, Ocean, and Coastal Science and Information Group, Leesburg, VA 20175, USA. [5]Dragonfly Data Science, 158 Victoria St, Level 4, Te Aro, Wellington 6011, New Zealand. [6]Center for Global Discovery and Conservation Science, Arizona State University, Tempe, AZ 85281, USA.

[*]Corresponding author. Email: jw2@hawaii.edu

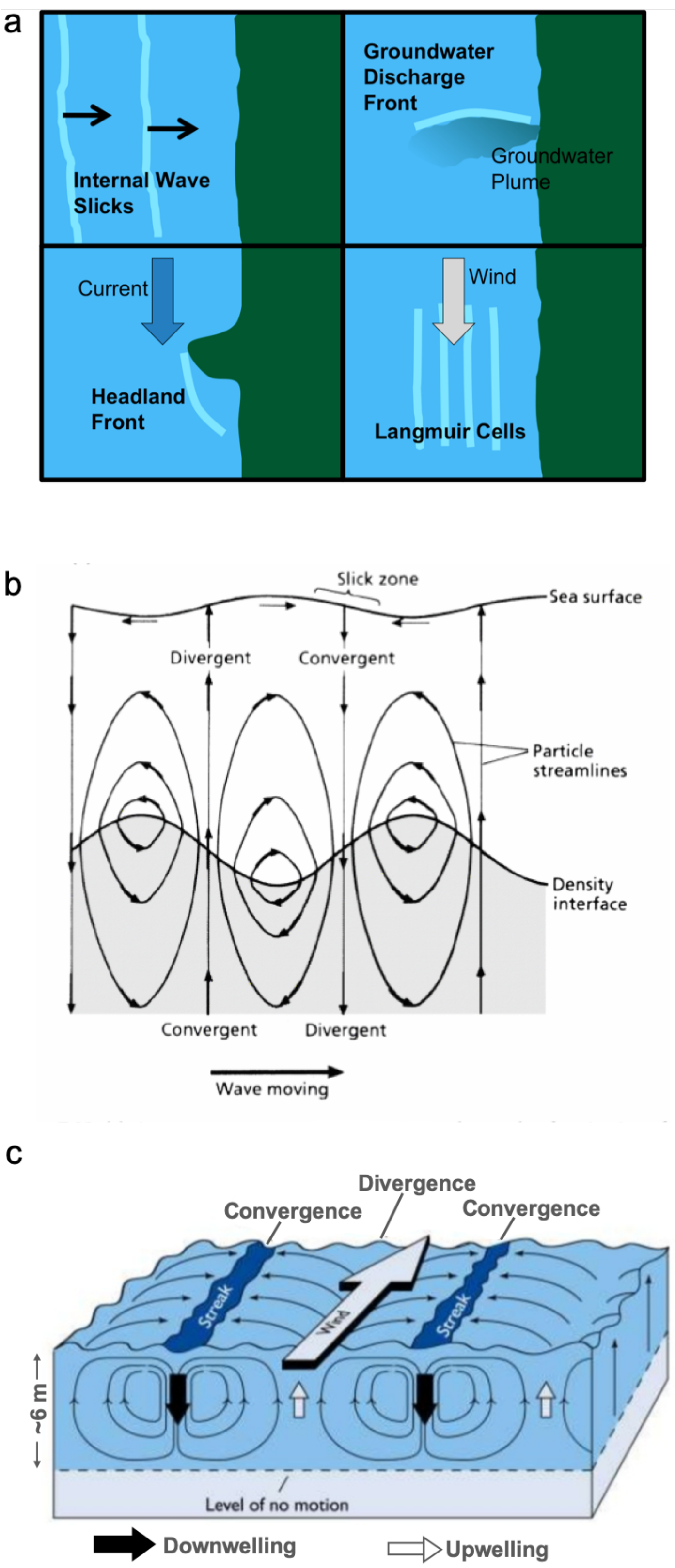


**Supplementary Fig. S1 | Physical mechanisms underlying surface slick formation and examples of convergent surface flow.** (**a**) Examples of physical mechanisms responsible for generating slicks in our study area: Internal wave slicks, groundwater discharge plume front, headland front, Langmuir cells. Each slick surveyed in the field study was categorized according to its most probable generating mechanism, considering all field observations and available supplementary data. These data included the GPS mapping of the surveyed slicks, field notes, wind conditions, tidal phase, maps of submarine groundwater discharge (SGD), bathymetry, SAR imagery, images from Landsat 8 and Sentinel-2, persistence and distance from shore. (**b**) Cross-section of an interfacial internal wave depicting currents associated with the propagating wave. While internal wave slicks may be located over either the convergent or divergent surface flows associated with internal waves, depending on whether surfactants or straining, respectively, are the dominant cause of ripple suppression, in this contribution we discuss convergent slicks. Reprinted with permission from Mann & Lazier [[1]]. (**c**) Conceptual diagram of surface flow circulation using example from Langmuir cell. Cross-section illustrates how physical driver (in this case wind) generates zones of convergent and divergent flow. Surface water moves from regions of divergence (over upwelling water) into zones of convergence (over downwelling water), creating a visible manifestation at the surface (streak or slick). The downwelling convergent flow can accumulate floating debris and upward swimming animals in the slick. Modified with permission from Tejada-Martínez et al. [[2]].

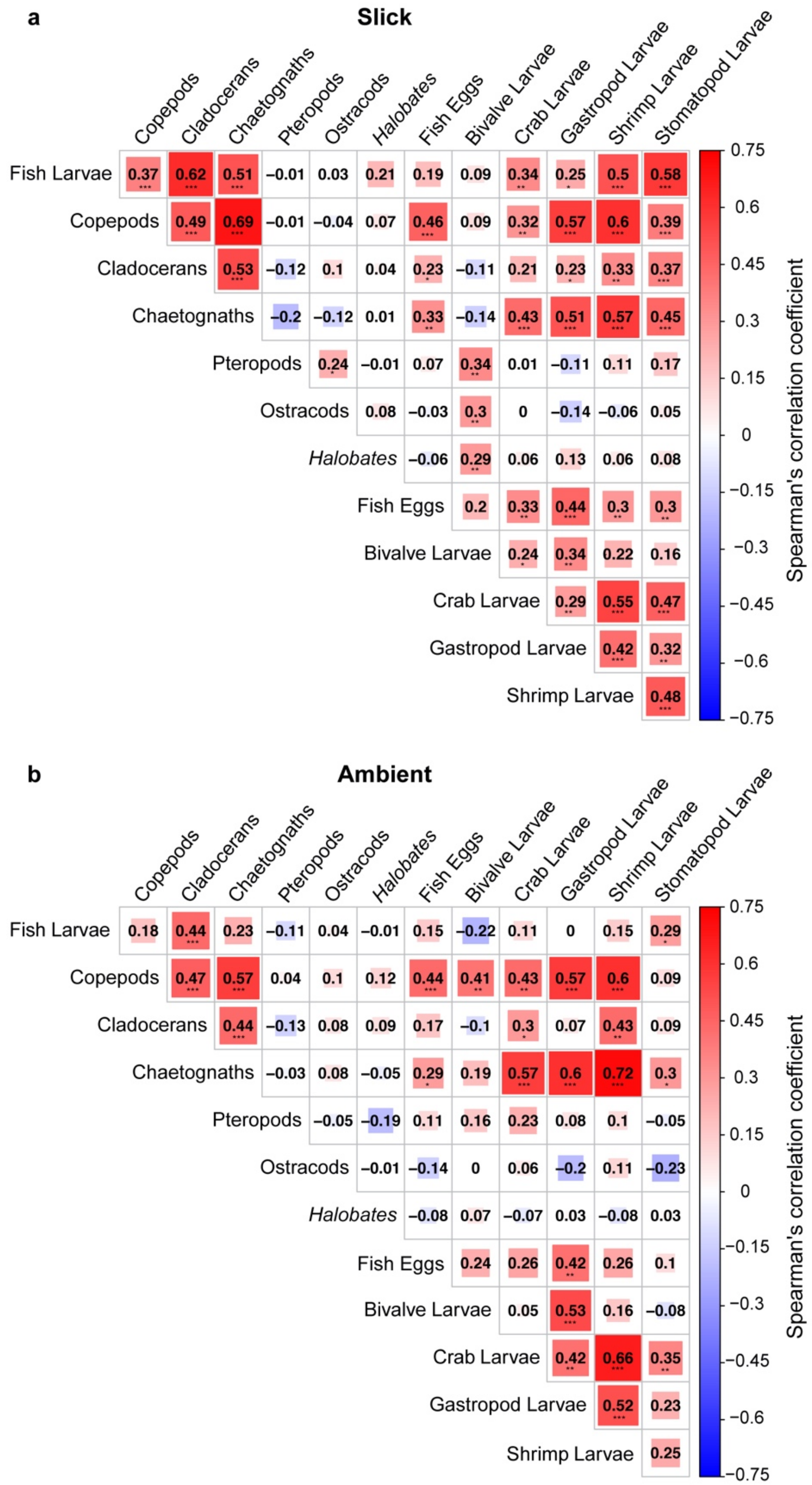


**Supplementary Fig. S2 | Spearman correlation heatmaps for densities of fish larvae and zooplankton prey groups in (a) surface slicks and (b) ambient water.** Correlation coefficients measure the strength of association between two taxa using log densities (individuals $m^{-3}$ per tow) and are displayed in each matrix along with significance levels (* < 0.05, ** < 0.01, *** < 0.001). The color and size of the square indicate strength of correlation: hotter (red) colors indicate higher positive correlations, colder (blue) colors indicate negative correlations, and white represents correlations near zero. The size of the square also indicates strength of correlations, with larger squares representing higher correlations.

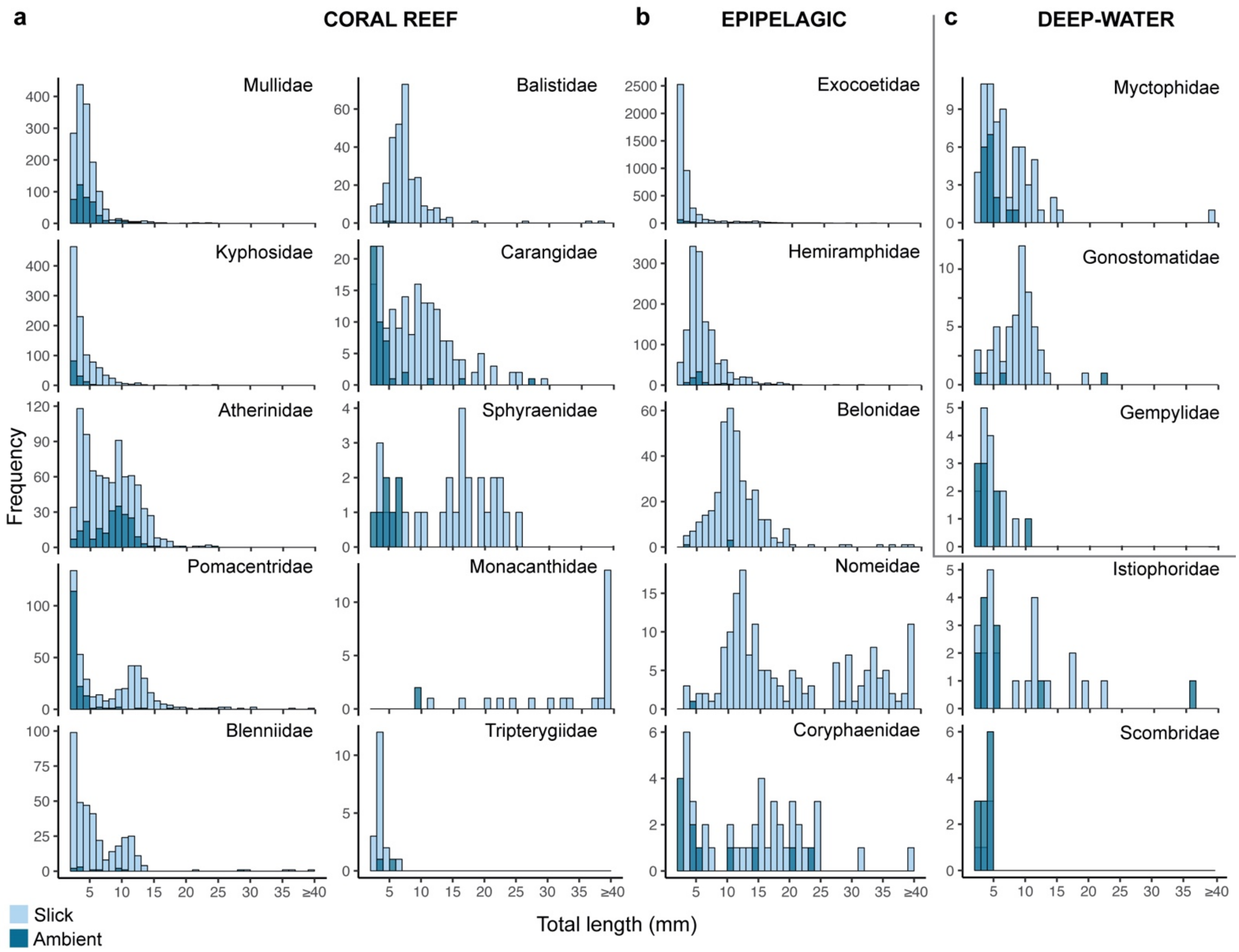


**Supplementary Fig. S3 | Histograms comparing size frequency distributions of larval fish families in surface slicks and ambient waters.** Data are presented for the 20 most abundant families (i.e., those present in ≥ 4 tows, and ≥ 15 individuals total), frequency of individual fish recorded in slicks (light blue) and ambient waters (dark blue). (**a**) Coral Reef fishes. (**b**) Epipelagic fishes. (**c**) Deep-water fishes.

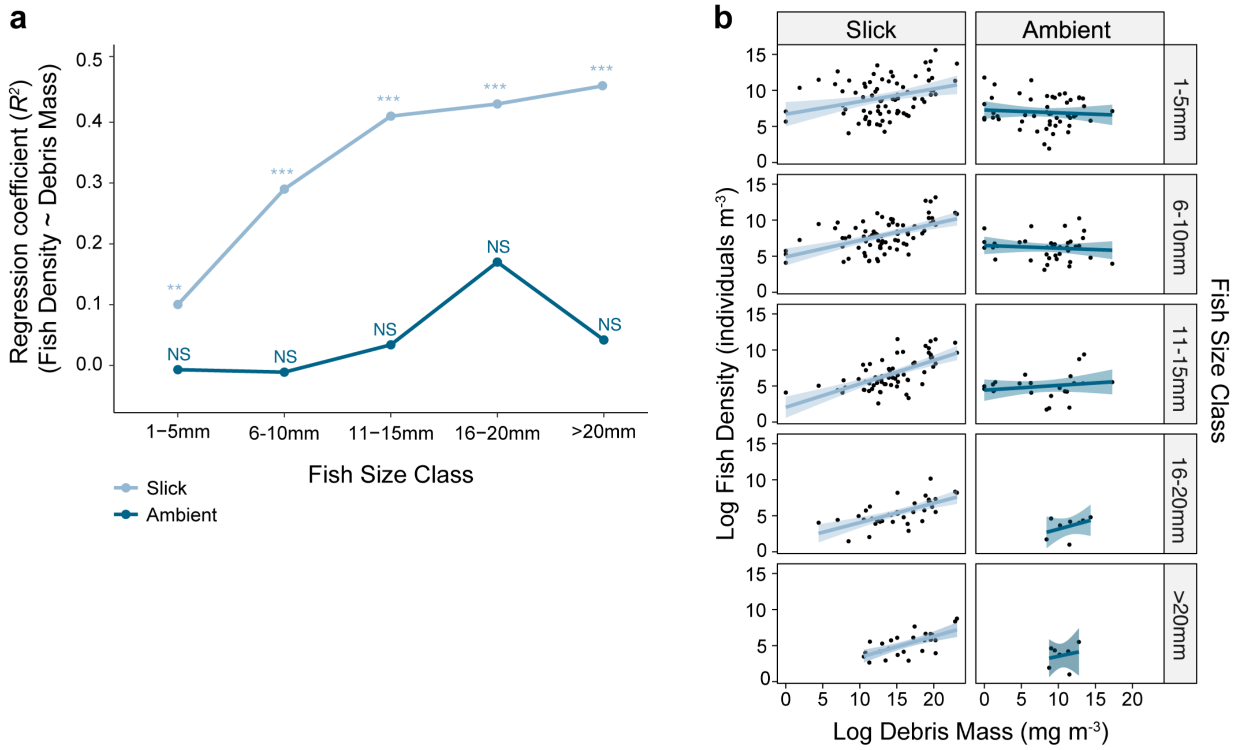


**Supplementary Fig. S4 | Relationship between fish density and floating debris as a function of fish size in surface slicks and ambient water habitats.** (**a**) Regression coefficients ($R^2$, y-axis) representing the linear relationship between log-transformed fish density (individuals m$^{-3}$; excluding zero densities) and log-transformed debris mass (mg m$^{-3}$, organic and plastic) for five size-classes of larval fishes (x-axis) in 5-mm bins (TL: <5mm, 6-10mm, 11-15mm, 16-20mm, >20mm). These relationships were explored separately in surface slicks (light blue) and ambient water (dark blue). The significance of the linear regression for each size-class and habitat is indicated (NS = Not significant $P > 0.05$; * = $P < 0.05$; ** = $P < 0.01$; *** = $P < 0.001$). (**b**) Scatterplots of log-transformed fish density (y-axis) and log-transformed debris mass (x-axis, mg m$^{-3}$, organic and plastic) for five size-classes of larval fishes (as above) in both slick (left) and ambient (right) habitats.

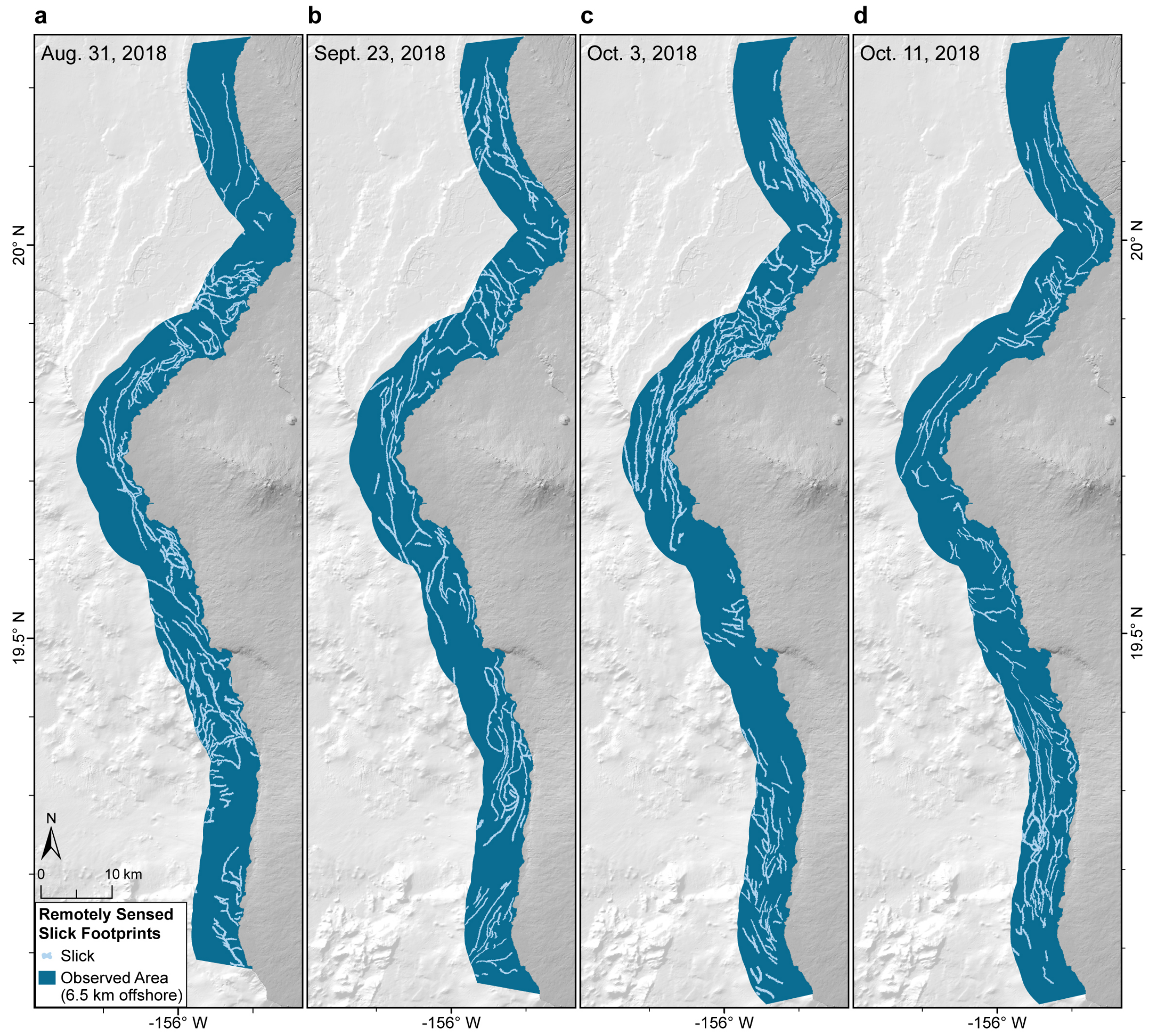


**Supplementary Fig. S5 | Remotely sensed surface slicks along the west coast of Hawai'i Island**, shown for 31 August 2018 (**a**), 23 September 2018 (**b**), 03 October 2018 (**c**), and 11 October 2018 (**d**). The spatial extent of remote sensing detection is shown as shaded regions in each panel and are constrained to the spatial extent of our neuston plankton samples (≤ 6.5 km). Maps were produced with ArcGIS Desktop 10.6 software (https://desktop.arcgis.com/), utilizing Planet Dove satellite images (https://www.planet.com) and a mapping approach that classifies slicks using the contrast between surface texture of slicks and regular ambient seawater (see *Methods – Remote Sensing* and Gove & Whitney et al. [3] for details).

**Supplementary Note 1: Water striders (*Halobates* spp.)**
We provide the first evidence that marine water striders (*Halobates* spp.) are strongly associated with slicks. *Halobates* adults were 6.6 times more abundant in slicks ($P(\bar{d}_{slick} > \bar{d}_{ambient}) \geq 0.9999$), and densities of nymphs were 4.3 times higher ($P(\bar{d}_{slick} > \bar{d}_{ambient}) = 0.97$; Supplementary Table S2). The majority (71.1%) of adults were *Halobates hawaiiensis*, and the minority *H. sericeus*. *H. hawaiiensis* were found primarily in slicks and mean densities were 6.9 times higher in slicks ($P(\bar{d}_{slick} > \bar{d}_{ambient}) \geq 0.9999$). *Halobates* principally feed on terrestrial insects blown out to sea [4], which were 26.6 times more concentrated in slicks ($P(\bar{d}_{slick} > \bar{d}_{ambient}) \geq 0.9999$). *Halobates* are prey to a number of juvenile fishes including jacks (Carangidae), chubs (Kyphosidae), damselfishes (Pomacentridae), and triggerfishes (Balistidae)[5], all of which were densely concentrated in slicks. *Halobates* are also found in the diets of many neustonic-feeding seabirds including some species of shearwater that feed on them exclusively [6]. Therefore, enrichment by insect bodies in slicks along with the water striders that eat them ultimately enhances feeding rates both above and below the surface.

**Supplementary Note 2: Diversity within fish families**
The three most diverse families accumulating in slicks were flyingfishes (11 species), jacks (10 species) and goatfishes (6 species; Supplementary Table S4). For flyingfishes, larvae of all 11 species were at least 6.8 times more abundant in slicks, including five species found exclusively in slicks. Nine species of jacks were at least 4.1 times more abundant in slicks including large bodied predators *Caranx* spp. (*C. ignobilis, C. melampygus*, and *C. sexfasciatus*), *Seriola* spp. (*S. dumerilli* and *S. riviolana)*, *Scomberoides lysan*, and *Pseudocaranx cheilio* as well as the coastal schooling pelagic *Decapterus macarellus* (Supplementary Table S4).

Larval fish of the order Beloniformes (flyingfishes, halfbeaks, and needlefishes) were almost exclusively concentrated in slicks. Three genera of flyingfish larvae were found exclusively in slicks (*Cypselurus, Hirundichthys*, and *Prognichthys*), and three additional genera (*Cheilopogon*, *Exocoetus*, and *Paraexocoetus*) were highly concentrated in slicks (6.8 - 35 times; $P(\bar{d}_{slick} > \bar{d}_{ambient}) \geq 0.9999$; Supplementary Table S4). Three of four locally occurring species of needlefishes (*Ablennes hians*, *Platybelone argalus*, and *Tylosurus acus melanotus*) were exclusive to slicks and the fourth (*Tylosurus crocodilus*) was 86 times more abundant in slicks. All four locally occurring halfbeak species (*Euleptorhamphus viridis*, *Hemiramphus depauperatus*, *Hyporhamphus acutus pacificus*, and *Oxyporhamphus micropterus*) were between 2 and 213 times more abundant in slicks (Supplementary Table S4).

**Supplementary Note 3: Sampling biases and potentially confounding variables**
ANOVA results indicate that sampling order, gear type, year or day had no significant effect on log response ratios for any of the functional groups tested: larval fishes ($F_{1,47} < 0.65$; $P > 0.42$), juvenile fishes (TL > 20mm; $F_{1,46} < 0.22$; $P > 0.64$), debris ($F_{1,41} < 0.96$; $P > 0.33$), and zooplankton ($F_{1,47} < 3.23$; $P > 0.07$). Therefore, we conclude that these effects explain little of the variation in response ratios, and do not represent significant confounding variables. Regarding potential net avoidance, we found there was no significant relationship between time of day (minutes from solar noon) and log response ratios of total fish density ($F_{1,22} = 1.95$; $R^2 = 0.018$, $P = 0.165$) or juvenile fish density (TL > 20mm; $F_{1,22} = 0.01$; $R^2 = -0.05$, $P = 0.92$). Therefore, we do not see a relationship between response ratios and time of day that would be indicative of a pattern driven by net avoidance. In addition, we performed an ANCOVA with log debris mass (mg $m^{-3}$) and time (minutes from solar noon) as covariates and habitat (slick vs. ambient) as the factor on all 134 transects (n = 80 slicks, n = 54 ambient tows). We found that debris mass has a significant effect on total fish density ($F_{1,128} = 11.3$; $P < 0.0001$), time of day (minutes from solar noon) did not have an effect ($F_{1,128} = 1.74$; $P = 0.1$), and once these covariates were accounted for, there remained a

significant effect of habitat (slick:ambient; $F_{1,128} = 33.7$; $P < 0.00001$). These results are consistent when tested for small larvae (TL<10mm) and large larvae (TL>10mm). For example, we found that debris mass has a significant effect on fish density for large larvae (TL>10mm; $F_{1,91} = 16.6$; $P < 0.00001$), and once accounted for, there remains a significant effect of habitat (slick:ambient; $F_{1,91} = 12.8$; $P < 0.001$).

## References


1. Mann, K. H. & Lazier, J. R. N. *Dynamics of Marine Ecosystems: Biological-Physical Interactions in the Oceans*. (Wiley-Blackwell, 2006).
2. Tejada-Martínez, A. E., Akkerman, I. & Bazilevs, Y. Large-Eddy Simulation of Shallow Water Langmuir Turbulence Using Isogeometric Analysis and the Residual-Based Variational Multiscale Method. *Journal of Applied Mechanics* **79,** 010909–1–010909–12 (2011).
3. Gove, J. M., Whitney, J.L., McManus M.A., Lecky J., Carvalho F.C., Lynch J.M., Li J., Neubauer P., Smith S., Phipps J.E., Kobayashi D., Balagso K.B., Contreras E.A., Manuel M.E., Merrifield M.A., Polovina J.J., Asner G.P., Maynard J.A., Williams G.J. Prey-size plastics are invading larval fish nurseries. *Proc Natl Acad Sci U S A* **53,** 201907496 (2019).
4. Cheng, L. Notes on the ecology of the oceanic insect *Halobates*. *Mar Fish Rev* (1974).
5. Senta, T., Kimura, M. & Kanbara, T. Predation of fishes on open-ocean species of sea-skaters (*Halobates* spp.). *Japanese Journal of Ichthyology* **40,** 193–198 (1993).
6. Harrison, C. S., Hilsa, T. S. & Seki, M. P. Hawaiian seabird feeding ecology. *Wildlife Monographs* 3–71 (1983).
